# Remote triggering of high magnitude earthquakes along plate boundaries

**DOI:** 10.1038/s41598-022-05102-4

**Published:** 2022-01-21

**Authors:** Robert T. O’Malley, Ayush Choudhury, Yue Zhang

**Affiliations:** 1 Department of Botany and Plant Pathology, Cordley Hall 2082, Oregon State University, Corvallis, OR 97331-2901 USA; 2 School of Electrical Engineering and Computer Science, 3117 Kelley Engineering Center, Oregon State University, Corvallis, OR 97331 USA

**Keywords:** Geology, Seismology

## Abstract

It has been shown that large magnitude earthquakes can remotely trigger other large magnitude earthquakes within three days. Such triggering of high magnitude earthquakes is potentially indicative of fault systems at the end of their seismic cycles. Here a method is developed to examine local earthquake history to determine how susceptible a given area is to remote triggering of high magnitude earthquakes. The method is applied to all plate boundaries. Only 14% of global tectonic boundaries are not susceptible to remote triggering, while 86% show susceptibility to varying degrees. The most highly susceptible locations begin triggering at lower magnitudes, dependent on the type of plate boundary. Varying patterns in susceptibility to remote triggering are observed around individual plates. Finite element modeling of the Cocos Plate reveals normal modes which appear consistent with its spatial patterns of high susceptibility. Many of the natural frequencies of the Cocos Plate are closely associated with the frequencies of free oscillations of the earth and could be induced by large earthquakes. Analysis of the stress tensors generated by the normal modes supports a delayed triggering mechanism involving one-sided negative (compressive) stress normal to the plane of the fault.

## Introduction

In order for high magnitude earthquake triggering to take place, there is a common assumption that the local fault systems are close to the end of their seismic cycle^1,2^. Such cycles can be on the order of less than a decade^3,4^ to many centuries^5,6^. In general, the magnitudes of these end-of-cycle events refer to magnitudes greater than the upper corner moment magnitude (Mc) of the modified Gutenberg–Richter formula describing the frequency distribution of earthquake magnitudes at a given locale^3,4^.

1 $$N\left(M\right)={N}_{0}{\left(\frac{{M}_{0}}{M}\right)}^{\beta }exp\left(\frac{{M}_{0}-M}{{M}_{C}}\right)$$

This equation gives the cumulative number of earthquakes for a given locale as a function of M (seismic moment or equivalent magnitude). Magnitudes below M_C_ fit the general Gutenberg–Richter linear relationship in a log–log plot (with beta as the slope; N_0_ and M_0_ associate with a minimum count and magnitude having complete coverage), while those above M_C_ show a drop off in frequency^7^. Earthquakes taking place in the linear portion of the curve below M_C_ are considered to be controlled by local forces, while discussions of periodicity and seismic cycles refer to earthquakes with magnitudes above M_C_^3^.

The implication of M_C_ as presented by McGuire^3^ would seem to be counter-intuitive. On the one hand it is accepted that triggered events are indicative of fault systems at the end of their seismic cycles, while on the other hand the presence of M_C_ implies those triggered earthquakes have to be large earthquakes (with magnitudes at or above M_C_). Small and mid-magnitude earthquakes inherently do not result from this process, only large events do. Up until 2018 there were few observations of high-magnitude triggering to support this conclusion. Such a view of triggered events, however, is consistent with recent global observations of remotely triggered high magnitude earthquakes^8^. In that paper, average global patterns connecting high magnitude source events and the resulting high magnitude triggered events were presented.

While the results of O’Malley et al.^8^ apply on a global scale, the extent of remote triggering that is possible at any specific location is unknown. Remote triggering of high magnitude events is controlled by local fault systems along plate boundaries and their inherent seismic cycles. If the likelihood of remote triggering of high magnitude earthquakes can be determined and mapped for specific areas, then we would gain insight into the distribution of risk associated with high magnitude remote triggering. Rather than doing fault-specific analyses, we will develop a regional analysis to detect the presence of this type of high magnitude triggering. Such an approach could be used to investigate all tectonic boundaries around the world. Any observations of spatial patterns of remote triggering would necessarily test our understanding of the underlying processes along tectonic boundaries.

### Previous work

General findings on remotely triggered high magnitude earthquakes^8^ indicate that triggered events are seen to be preceded within 3 days by large magnitude earthquakes (magnitudes ≥ M6.0) up to 180° away, and only the largest magnitude events are being triggered. These conclusions were drawn after the removal of small magnitude events from the input data, along with the removal of data from aftershock sequences (Methods). Both small-magnitude triggering and aftershock sequences are known triggering processes, and their effects were removed from the study. We shall use the approach and findings of that paper as the basis and starting point for this paper. It is noted that the 3-day time period for triggering gave the strongest global results, and will be used in this analysis as well.

In the O’Malley et al.^8^ paper, the observation is made that there are ‘source’ events that initiate the delayed triggering sequence. Any earthquake of magnitude ≥ M6.0 is a potential source event. Any earthquake of magnitude ≥ M5.0 is a potentially triggered event, and it was noted that the USGS catalog used for the study was found to be complete at this level of detection. The time period that separates the source event from the triggered event was set at 72 h, although the impact may be longer for the highest magnitude source events.

In that study, aftershocks and foreshocks were removed from the USGS catalog using the windowing technique applied by Gardner and Knopoff^9^ (Methods). While there were statistical reasons for applying that method to decluster the data, the practical result was to avoid biasing the results with aftershock and foreshock data. Also, when we speak of remote triggering by source events, ‘remote’ is effectively any distance beyond the aftershock zone of the source event.

Two different numerical experiments were applied in that paper: one that looks forward in time, starting with potential source events, and one that looks backward in time starting with potentially triggered events. Both approaches indicated the presence of systematic triggering of medium to high magnitude earthquakes by high magnitude source events. In this paper we will use the second approach, starting with potentially triggered events.

The variable that was analyzed in that study was the p-value (Methods). Most are familiar with using p-values to give an estimate of how well data fit a theoretical curve, etc. In this case, however, it is appropriate to think of it as a basic calculation of probability. What are the odds of getting 5 heads in 7 coin tosses when there’s a 50% chance of getting heads? What about 15 in 21, or 20 in 28? The p-value keeps dropping even though the ratio of success remains the same, and is a function of sample size. This is the calculation being applied here.

In this study, the set of potentially triggered events is equivalent to the number of coin tosses. Observations of the number of ‘heads’ becomes the number of times we find one or more source events preceding the potentially triggered event within 72 h. And instead of assuming a ‘fair’ coin toss (with a probability of exactly 0.50 of tossing heads or tails) we examine the declustered catalog to find how often potentially triggered events (M ≥ M5.0) are preceded within three days by one or more potential source events (M ≥ M6.0). Surprisingly, this ratio is almost exactly equal to 50% (prob = 0.501; Supplemental Information, Section 1). Everything we know about coin tosses applies here.

In this paper we shall begin with the potentially triggered events and look backwards in time for the source events. Rather than doing a global analysis of all triggered events, we shall proceed with a local set of events that fall within a given study area. And the p-value (based on the number of potentially triggered events preceded by source events, given prob = 0.501) becomes the variable of interest.

### Methodology—basic approach

We begin with the declustered, global USGS catalog for all earthquakes of magnitude ≥ M5.0 from O’Malley et al.^8^. Those earthquakes are then compared with the results calculated by the Global Centroid-Moment-Tensor (CMT) Project^10,11^. When matchups are available, the strike, dip and rake of the primary focal plane are extracted. The rake is broadly used to classify the type of faulting. A rake of 0° ± 30°, or 180° ± 30° was defined as a strike-slip fault. A rake of − 90° ± 60° is a normal fault (where the hanging wall drops) and a rake of + 90° ± 60° is a reverse (or thrust) fault. Visual examination of a histogram of the accumulated rakes supports the three distinct populations.

All earthquakes of magnitude ≥ M5.0 that fall within a given project area are extracted from the catalog. This becomes the set of all potentially triggered earthquakes, and it can be analyzed collectively, or by type of faulting. The project area used in this paper (Methods) is a bounding box centered on a location of interest and extending ± 2.85° to the side, normalized for latitude (~ 100,000 km^2^ per quadrant).

Each of the potentially triggered earthquakes (M ≥ M5.0) is then examined to see if there exists one or more source events (M ≥ M6.0) occurring in the previous 72 h from anywhere on the globe. The result is that we have ‘x’ observations of one or more source events for ‘n’ potentially triggered earthquakes. We also know the probability of seeing one or more source events within 72 h of a potentially triggered event (prob = 0.501). Given ‘x’, ‘n’, and ‘prob’ we can directly apply the binomial theorem to obtain the p-value. In MATLAB^12^ this is found using the call for the binomial cumulative distribution function (***binocdf***). As a note, we will be using the ‘mid’ p-value^8^, which is the average of the p-values calculated using ‘x’ and ‘x − 1’.

It is possible to apply this method in a manner that indicates how the p-value changes based on magnitude for a given study area (Methods). For example, if we put in all potentially triggered events, we will have an estimate of the p-value for all earthquakes with a lower bound of M5.0 and covering all earthquakes up to the largest observed magnitude. We could then repeat the calculation, but now increasing the lower bound to M5.1. Then do it again, now starting with a lower bound at M5.2, etc., until it gets up to the maximum magnitude for the area. What results is a p-value curve as a function of the lower bound magnitude. This p-value curve can be analyzed to determine (1) how susceptible the area is to remote triggering of high magnitude earthquakes, and (2) what the onset magnitude is for this type of triggering to occur.

Imagine the case of a study area where Eq. (1) is expressed: where high magnitude earthquakes are being remotely triggered, but where local forces control the lower magnitude earthquakes (no remote triggering for those magnitudes). The high magnitude earthquakes will have relatively more source events present than the low magnitude set. The p-value calculated from M5.0 to the maximum magnitude will be higher, approaching 0.5 (or more) since it includes low magnitude events that are non-triggered. As the lower bound is increased, the number of non-triggered events will decrease (because the lowest magnitudes won’t be counted) and the p-value will start dropping. At some point the onset magnitude for remote triggering will be reached, and the majority of the non-triggered events will be excluded. With the lower bound set at the onset magnitude, the sample size will be the largest possible for the triggered events, and the p-value will be a minimum. This minimum in the p-value locates the onset magnitude for remote triggering, and the p-value for that lower bound indicates the susceptibility to remote triggering for that study area. Generally speaking, p-values at or above 0.5 indicate regions of no remote triggering, while regions with p-values below 0.05 will be considered highly susceptible to such triggering. In practice, there are locations where the p-values get much smaller than 0.05.

### Examples of local data analysis

**Figure 1:**
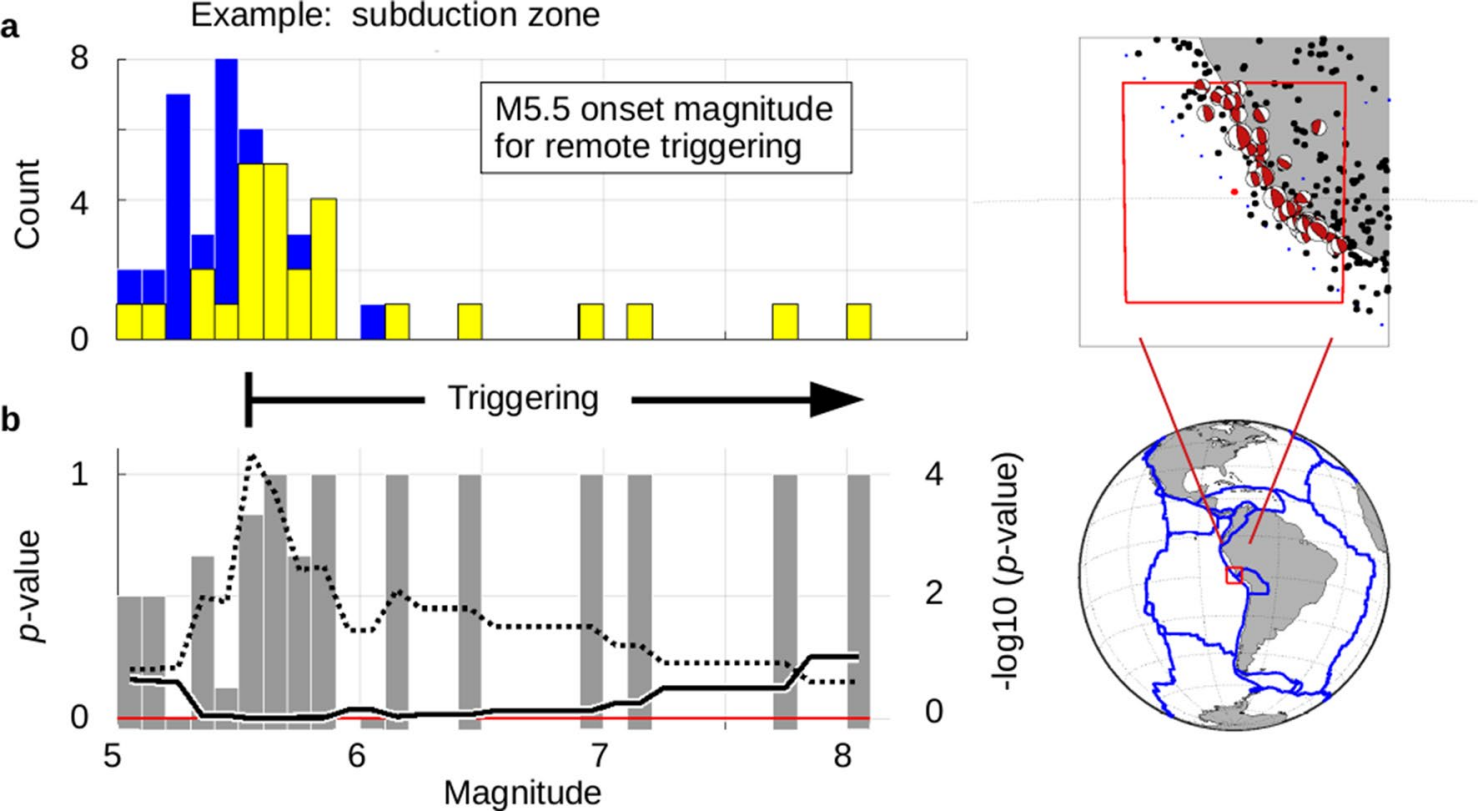
Local Detection of Remote Triggering of High Magnitude Earthquakes. The red box (index maps^12^) indicates the area under analysis (± 2.85°, normalized by latitude). The red dot indicates the center of the box. Black dots represent earthquakes ≥ M5.0 in the region, and red beach balls indicate the focal mechanisms for thrust events in the study area. Blue dots represent digital plate boundaries^15^. Magnitude bins are in tenths of a magnitude, ‘M5.0’ only includes data ≥ M5.0 but < M5.1, etc. (**a**) Earthquakes ≥ M5.0 within the target area are selected (after removal of aftershocks) and tallied by magnitude (blue bars). Those earthquakes that are preceded by one or more source events within 3 days are also tallied and overlain in yellow. (**b**) The ratio of earthquakes with source events (yellow) to total number of earthquakes (blue) for any given bin is shown in gray bars. The odds of seeing the combined yellow to blue counts given an average probability of 0.501 are calculated as a p-value curve (solid line) (see text). The dotted curve plots the − log10 transform of the p-value and locates the corner associated with the onset magnitude for triggering.

Consider the subduction zone off the coast of Peru, where the Nazca plate moves under the South American plate as well as the Altiplano microplate. The selected region is very nearly centered on the junction of these three plates (Fig. 1). The largest earthquake in this region was the magnitude M8.0 Pisco, Peru earthquake of August 15, 2007^13,14^. Significant damage took place at the nearby city of Pisco.

A total of 140 local earthquakes of magnitude ≥ M5.0 have occurred in the marked region over the past 44 years (excluding aftershocks). Of those 140 earthquakes, the CMT project has 48 focal mechanism solutions for thrust earthquakes. Given that this is a subduction zone, we will examine these thrust events for our example. The bounding box used to select earthquakes was ± 2.85°, normalized for latitude (~ 100,000 km^2^ per quadrant). The magnitude distribution is shown for these potentially triggered thrust events (Fig. 1a). The number of earthquakes in a given bin that were preceded by one or more source events within a three-day period is overlain in yellow. If only yellow is seen, then all events in that bin were preceded by source events. For magnitude M5.5 and larger, only three earthquakes do not have a preceding source event (22 out of 25 total have source events). The p-value for this to represent a random drawing is less than 0.0001 (Fig. 1b). The minimum of the resulting p-value curve is used to indicate the onset for remote triggering of high magnitude events. The onset magnitude is most easily seen by looking for the corner in the p-value curve when plotted as − log10(p-value) (dotted line).

For the boxed region off the coast of Peru, the triggering zone begins at M5.5 and has an associated p-value of < 0.0001 for all events from M5.5 up to the highest magnitude earthquake of the region. A p-value of exactly 0.0001 indicates that there is one chance in 10,000 that the observed pattern is simply a random expression of the general probability. This region has been highly susceptible to remote triggering of high magnitude thrust earthquakes over the past 40 + years. See Supplemental Information, Section 3 for the global distribution of the source events associated with these events.

**Figure 2:**
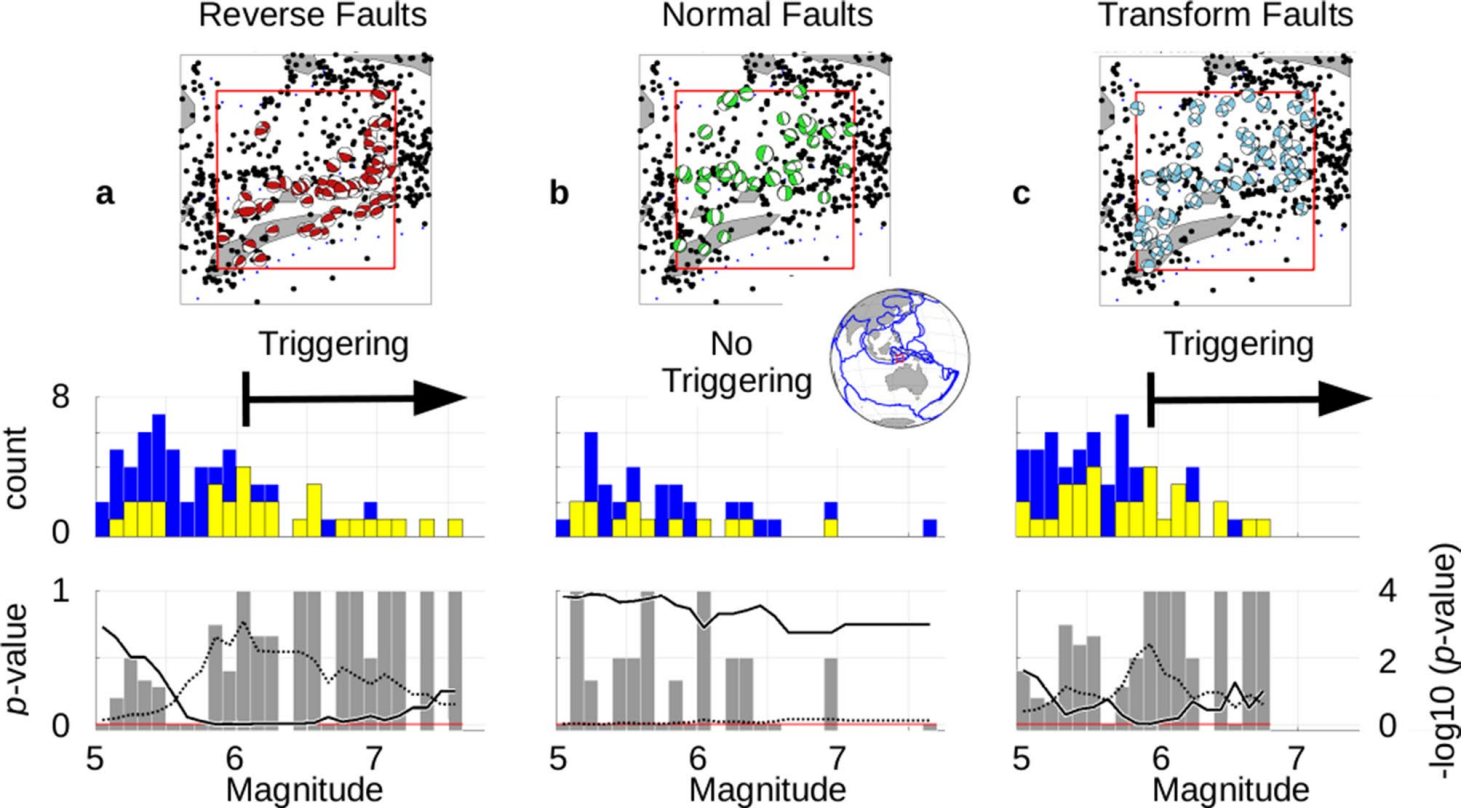
Differences in Triggering Based on Type of Faulting. The red boxes (index maps^12^) indicate the area under analysis (± 2.85°, normalized by latitude) for three different sets of earthquakes: (**a**) reverse, (**b**) normal and (**c**) transform. Focal mechanism are shown for each earthquake set. The blue lines on the map represent digital plate boundaries^15^. Magnitude bins for each set are in tenths of a magnitude, ‘M5.0’ only includes data ≥ M5.0 but < M5.1, etc. Earthquakes ≥ M5.0 within each target area are selected (after removal of aftershocks) and tallied by magnitude (blue bars). Those earthquakes that are preceded by one or more source events within 3 days are also tallied and overlain in yellow. The ratio of earthquakes with source events (yellow) to total number of earthquakes (blue) for any given bin is shown in gray bars. The odds of seeing the combined yellow to blue counts given an average probability of 0.501 are calculated as a p-value curve (solid line) (see text). The dotted curve plots the -log10 transform of the p-value and locates the corner associated with the onset magnitude for triggering. Reverse and transform faulting shows strong triggering, while normal faults are not being triggered in this locale.

For the next example we will consider a region identified as an oceanic convergence zone (Fig. 2). In this case we will examine the results for all three major types of faulting. As in the case for the subduction zone, earthquakes with reverse (thrust) faults (Fig. 2a) strongly indicate the presence of triggering, with 19 of 23 earthquakes at or above M6.0 having source events (p-value = 0.0008). However, earthquakes with normal faults do not exhibit any triggering at all for high magnitude events (Fig. 2b). The p-values for normal faulting in this zone of overall convergence never gets below 0.60. This implies that normal faulting is entirely controlled by local forces in this area. Transform faulting for the same region (Fig. 2c) shows the presence of triggering similar to reverse faulting, with 14 out of 17 earthquakes having source events at or above M5.9 (p-value = 0.0039). See Supplemental Information, Section 3 for the distribution of source events associated with Fig. 2a, c.

## Results for the plate boundaries

**Figure 3:**
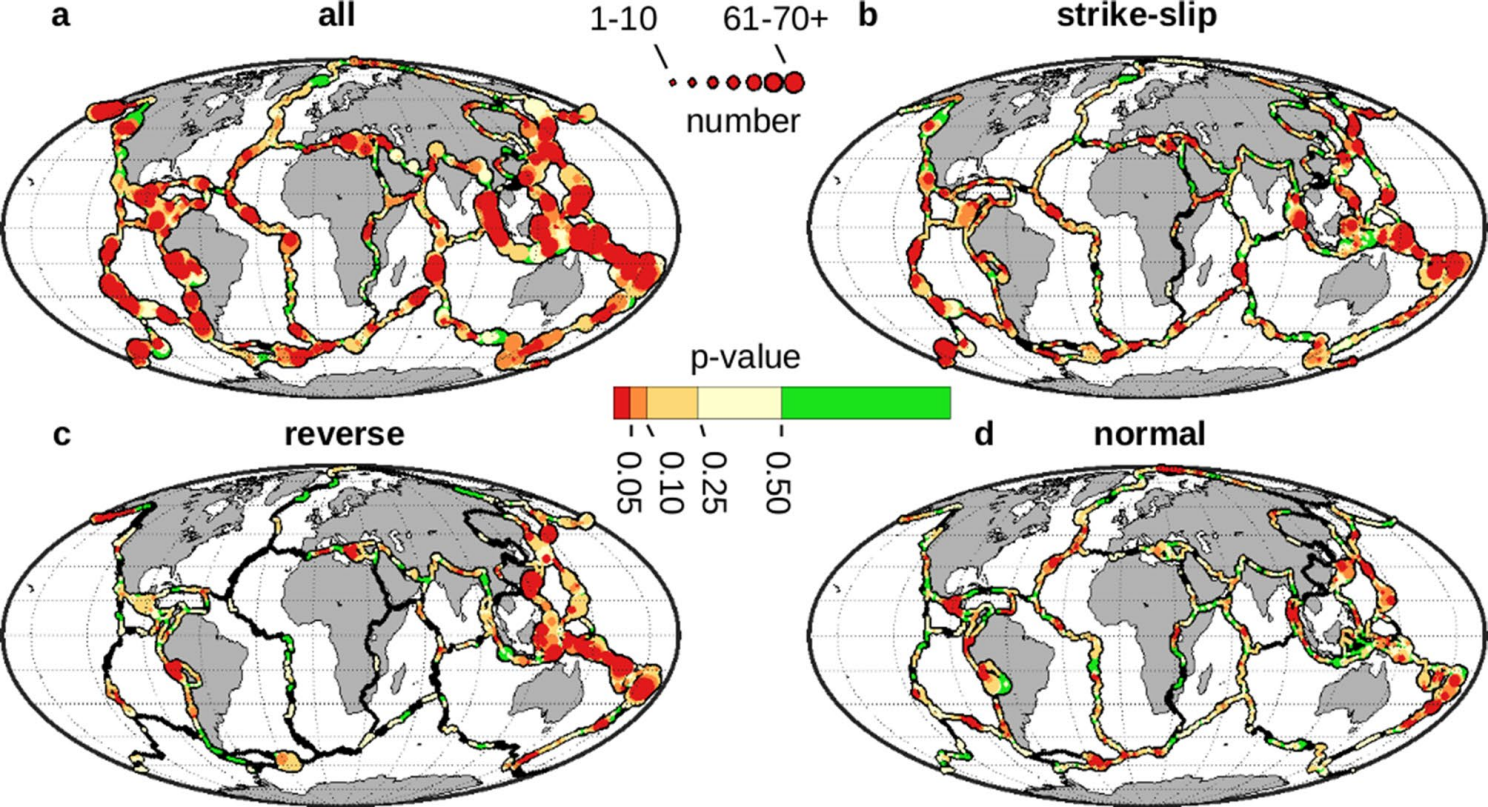
Plate boundaries susceptible to remote triggering of high magnitude earthquakes. Each location of the digital plate boundaries^15^ is locally tested for remote triggering of high magnitude earthquakes. The p-value for the triggering zone becomes the variable of interest and represents the susceptibility to this type of remote triggering. Green represents those areas with no susceptibility (p-values ≥ 0.50) while the highest level of susceptibility (red) is for p-values < 0.05. Black indicates no data. Line widths are scaled to the number of potentially triggered earthquakes at each location. (**a**) This is the global map^12^ that results when all earthquakes are combined and shows susceptibility to earthquakes taken collectively. (**b**) These are the results when only considering potentially triggered events with strike-slip faulting. They show a global prevalence to triggering of strike-slip faulting and represent many of the features seen in (**a**). (**c**) Susceptibility to triggering for reverse (thrust) faulting. (**d**) These are the patterns when only considering normal faulting for the potentially triggered events.

The above approach makes it possible to map the plate boundaries for their susceptibility to remote triggering of high magnitude earthquakes. The method can be applied systematically across the globe given digital locations that delineate the plates^15^. This can be done for all earthquakes combined, or for subsets based on focal mechanism (normal, reverse, or strike-slip earthquakes). The results show an extensive global presence of this type of delayed triggering (Fig. 3), whether analyzed collectively, or individually based on focal mechanism. Susceptibility to triggering is more pervasive for strike-slip than it is for for normal or thrust faulting. The p-values for strike-slip earthquakes are also more skewed to lower values than the other two types of faulting. The collective results (Fig. 3a) can be examined to test the global susceptibility to high magnitude triggering, regardless of focal mechanism. Plate boundary locations that do not exhibit this behavior (p-values ≥ 0.50; 14% of the locations) are shown in green, while 86% of the plate boundaries exhibit susceptibility to varying degrees. Those locations that are the most susceptible are shown in red, with p-values < 0.05 (19%).

**Figure 4:**
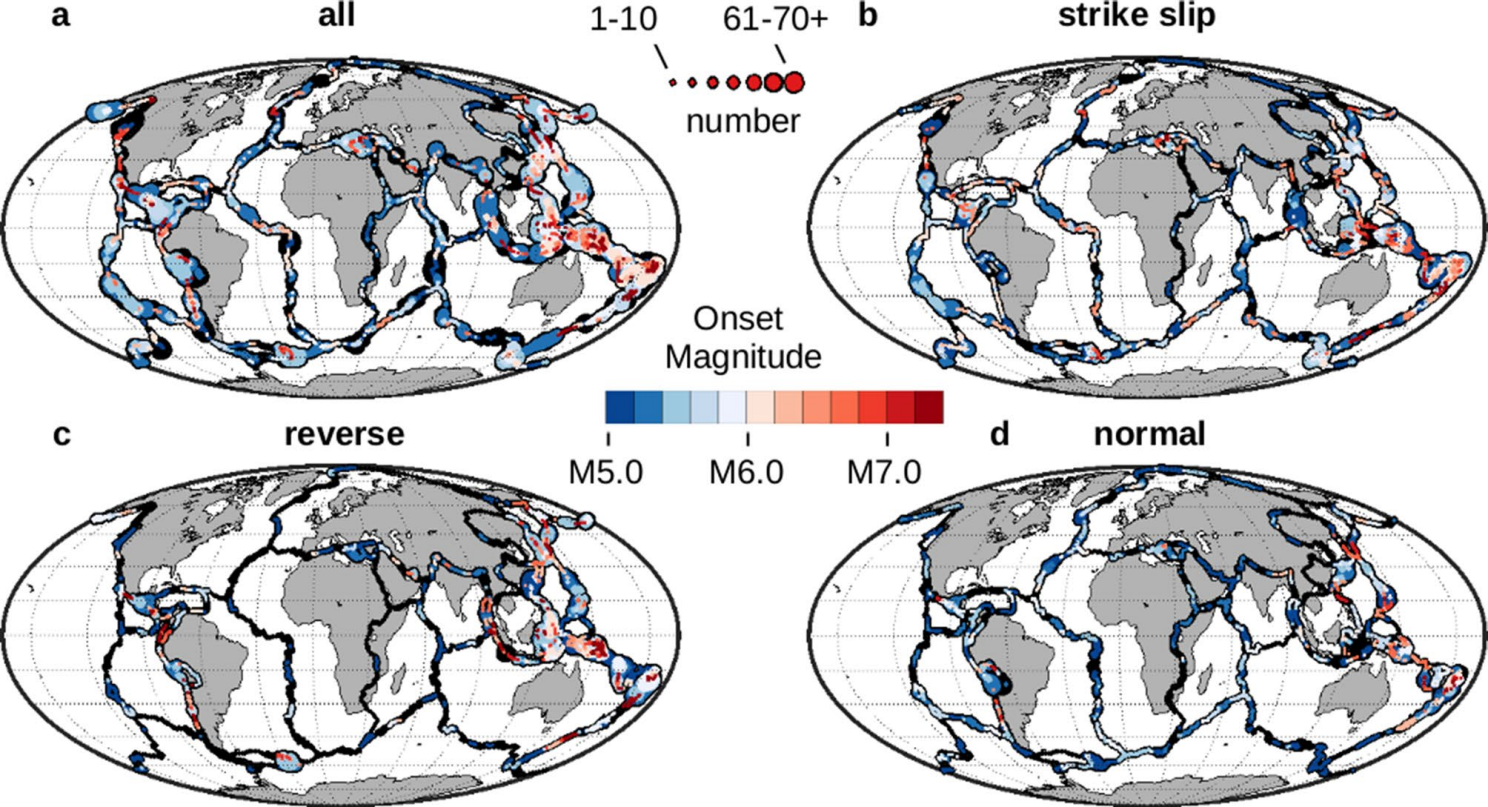
Onset magnitudes for remote triggering of high magnitude earthquakes. Each location of the digital plate boundaries^15^ is locally tested for remote triggering of high magnitude earthquakes. Global maps^12^ of the onset magnitude for this type of triggering are shown for different collections of potentially triggered earthquakes: (**a**) all combined, (**b**) strike-slip only, (**c**) reverse only, and (**d**) normal only. The onset magnitudes shown here are associated with the p-values shown in Fig. 3. Line widths are scaled to the number of potentially triggered earthquakes at each location. No onset magnitudes are shown for those locations that do not express this type of triggering (shown in black).

The associated onset magnitudes for remote triggering of high magnitude earthquakes are also available (Fig. 4). These results can also be obtained collectively, or based on focal mechanism. For the collective case, a majority of the plate boundaries (65%) are seen to have onset magnitudes that are below M6.0 (shown in blue).

The onset magnitude for remote triggering is potentially equivalent to the upper corner magnitude (M_C_) of the modified Gutenberg–Richter formula (Eq. 1). M_C_ is used in probabilistic estimates for regional seismicity^7,16,17^ and a variable proxy via the onset magnitude for remote triggering could be useful. The onset magnitude defined here is a new metric based on a regional analysis, and it delivers results based on data spanning 40 + years. For comparison, Boettcher and McGuire^4^ give M_C_ magnitude values for mid-ocean ridge transform faults of Gofar (segments G1, G2 and G3), and Discovery (segments D1 and D2). These five combined Gofar and Discovery segments in the East Pacific Rise are located within a single box in this study. The result found here was an onset magnitude of M6.1 for the strike-slip subset, which is comparable to the reported M_C_ values of M6.1, M5.8, M6.2, M5.6 and M6.0 for the individual segments. However, Boettcher and McGuire^4^ also report an M_C_ value of M6.4 for the Blanco Ridge, but here the best example that isolates the transform faults of the Blanco Ridge yields an onset magnitude that is much lower (M5.1).

**Figure 5:**
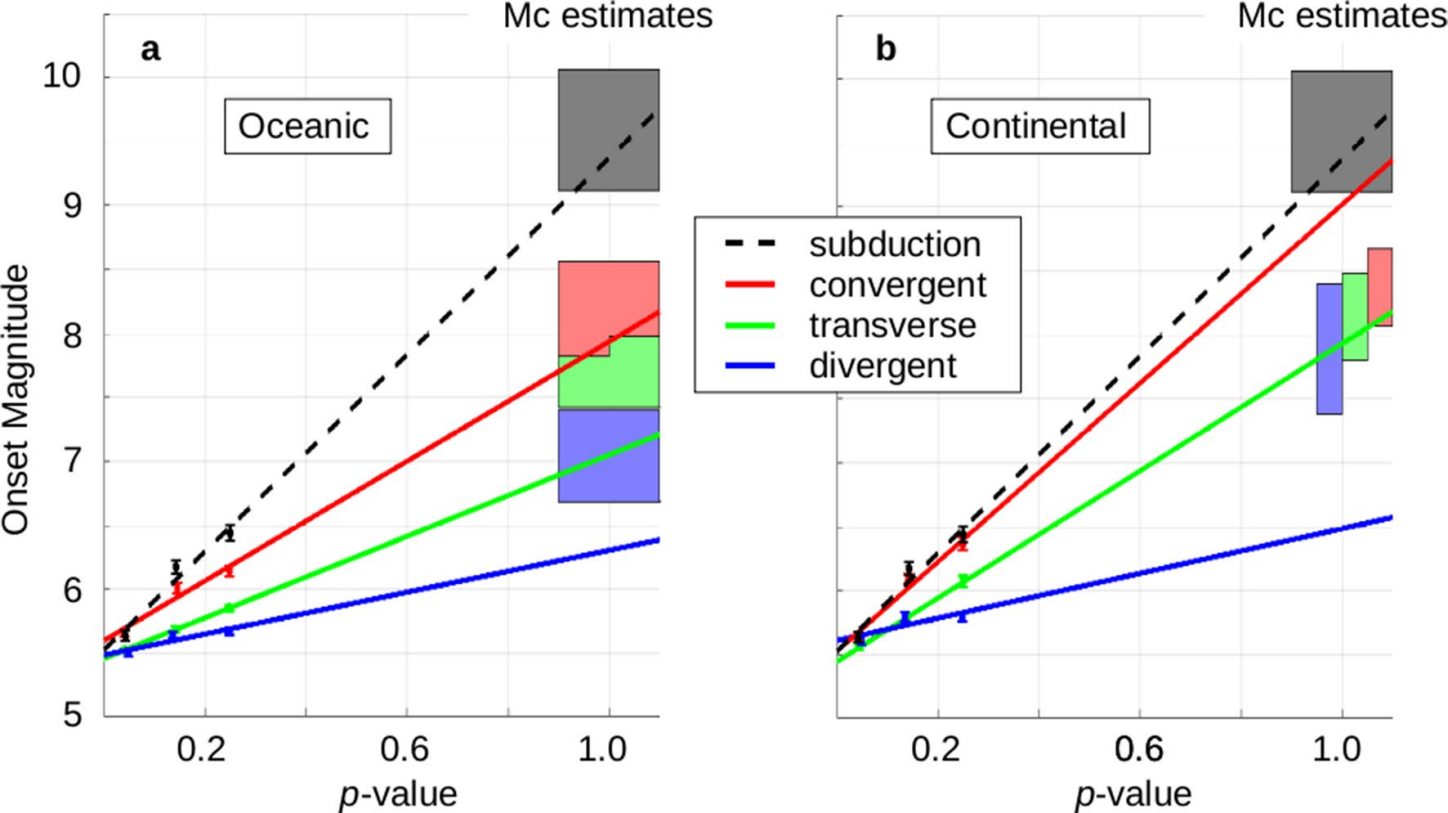
Onset magnitude as a function of remote triggering p-value. P-values were separated by the seven different boundary types and associated focal, then the onset magnitudes were binned as a function of p-value: 0.0–0.1, 0.1–0.2 and 0.2–0.3. The average for each bin is shown; error bars indicate the standard error to show how much variation in the average is possible. The results for subduction zones (black dashed line) gives the highest onset magnitude for all boundary types, and is shown on both plots for reference. (**a**) Results for oceanic boundary types (convergent, transverse and divergent). (**b**) Results for continental boundary types (convergent, strike-slip and divergent). Mc values^18^ are shown to illustrate the range found in their study.

Generalized M_C_ estimates are available for different tectonic settings, combining global statistics and model considerations^18^. They show that the M_C_ value for subduction zones is the largest of all possible plate boundaries. They also show that convergent boundaries have larger M_C_ values than divergent boundaries, and transform/strike-slip boundaries fall in between the two. However, their estimates of M_C_ are consistently greater than the onset magnitudes obtained here, and we will return to this in the Discussion section. For example, the highest onset magnitudes obtained in this study are M8.4, found along a segment where the Pacific Plate subducts under the Okhotsk Plate at 40° N latitude just east of Japan. Bird and Kagan^18^ report M9.58 (+ 0.48/-0.46) as representative of global subduction zones. Moreover, here the onset magnitude appears to be a function of the susceptibility to remote triggering: the higher the susceptibility the lower the onset magnitude (Fig. 5). The data were separated by plate boundary type and focal mechanism, and the onset magnitudes were averaged for three p-value bins (0.0–0.1, 0.1–0.2, and 0.2–0.3). Standard errors are shown to indicate the possible range in the calculated averages, and best fit lines were projected. The trace for the subduction zone is clearly greater than all other boundary types, and the order of convergent > transform > divergent is seen for both oceanic (Fig. 5a) and continental (Fig. 5b) plates. The colored boxes on the right of the plots show a range of M_C_ values resulting from Bird and Kagan’s^18^ work.

Given the global extraction of p-values and onset magnitudes based on focal mechanism, we were able to test the uniqueness of our observed results against synthetic (time-randomized) earthquake catalogs. We compared the value of the projected onset magnitude shown in Fig. 5 (at p-value = 1.0) with values obtained from synthetic catalogs for the seven different tectonic regimes (Supplemental Information, Section 2, Fig. 5). The observed data shown in Fig. 5 resulted in extreme values when compared with 64 of the 70 random results. The p-value associated with that test of randomness is on the order of 1e-14.

### Patterns of susceptibility and normal modes of the Cocos plate

**Figure 6:**
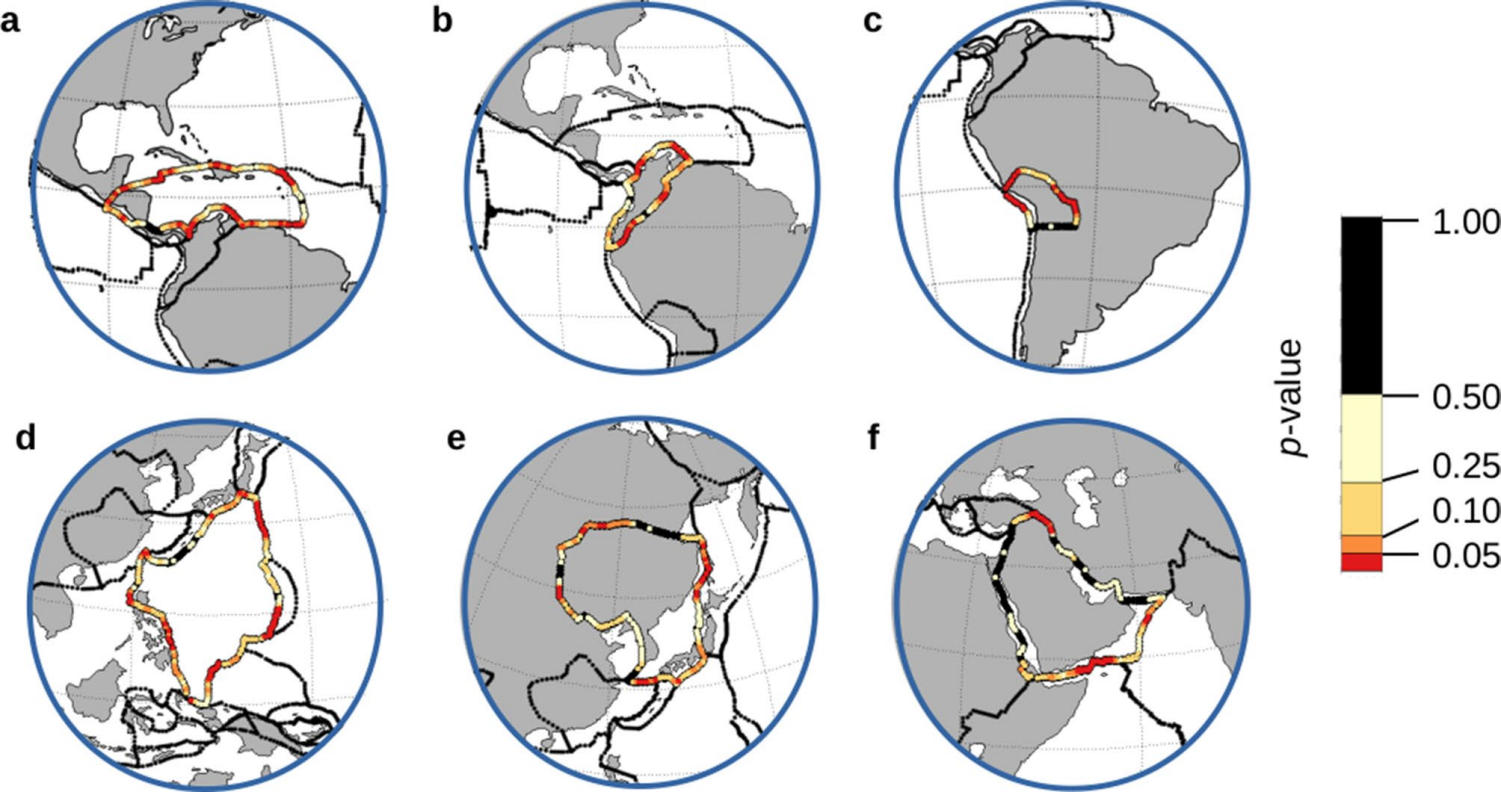
Spatial patterns of susceptibility to remote triggering around individual plates. Consistent patterns of highs and lows in susceptibility to triggering are seen surrounding individual plates^12^, while varying from plate to plate (data extracted from Fig. 3a). Constant p-value line widths were used to illustrate the patterns. (**a**) Caribbean plate. (**b**) North Andes plate. (**c**) Altiplano plate. (**d**) Philippine Sea plate. (**e**) Amur plate. (**f**) Arabia plate.

Local patterns of highs and lows in the susceptibility to remote triggering are present throughout the map of the combined earthquakes (Fig. 3a), from the tight, alternating pattern of the Caribbean plate to the broader spacing seen at the triple junction in the north Atlantic. Closer inspection shows that the local spacing appears to vary from plate to plate (Fig. 6). While similarities may be found, such as the simple two-high pattern surrounding the Altiplano plate (6c) and the Arabia plate (6f.), the lengths of the quiescent zones are much different. Similar comparisons can be made between Fig. 6a, d, as well as Fig. 6b, e. These patterns cross all plate boundary types. Spatial patterns in susceptibility which surround individual plates seem to imply some form of physical mechanism at play. If so, this unknown mechanism is somehow impacting the local susceptibility to remote triggering by modifying the boundary conditions, which includes lowering the onset magnitudes.

A potential mechanism that we will consider is thin-plate resonance. We check for two aspects to be present to verify if the normal modes of the plate should be considered as a possible factor impacting the boundary conditions along the edges of the plate. First, are there alignments of increased susceptibility with observed patterns in the various normal modes for the plate? If so, then the next becomes: what is driving the resonance? The answer to this will be found in the natural frequencies of the calculated normal modes. If there are global processes available with similar frequencies to drive the normal modes, then we have the potential for a mechanism to be involved with this type of high-magnitude delayed triggering.

**Figure 7:**
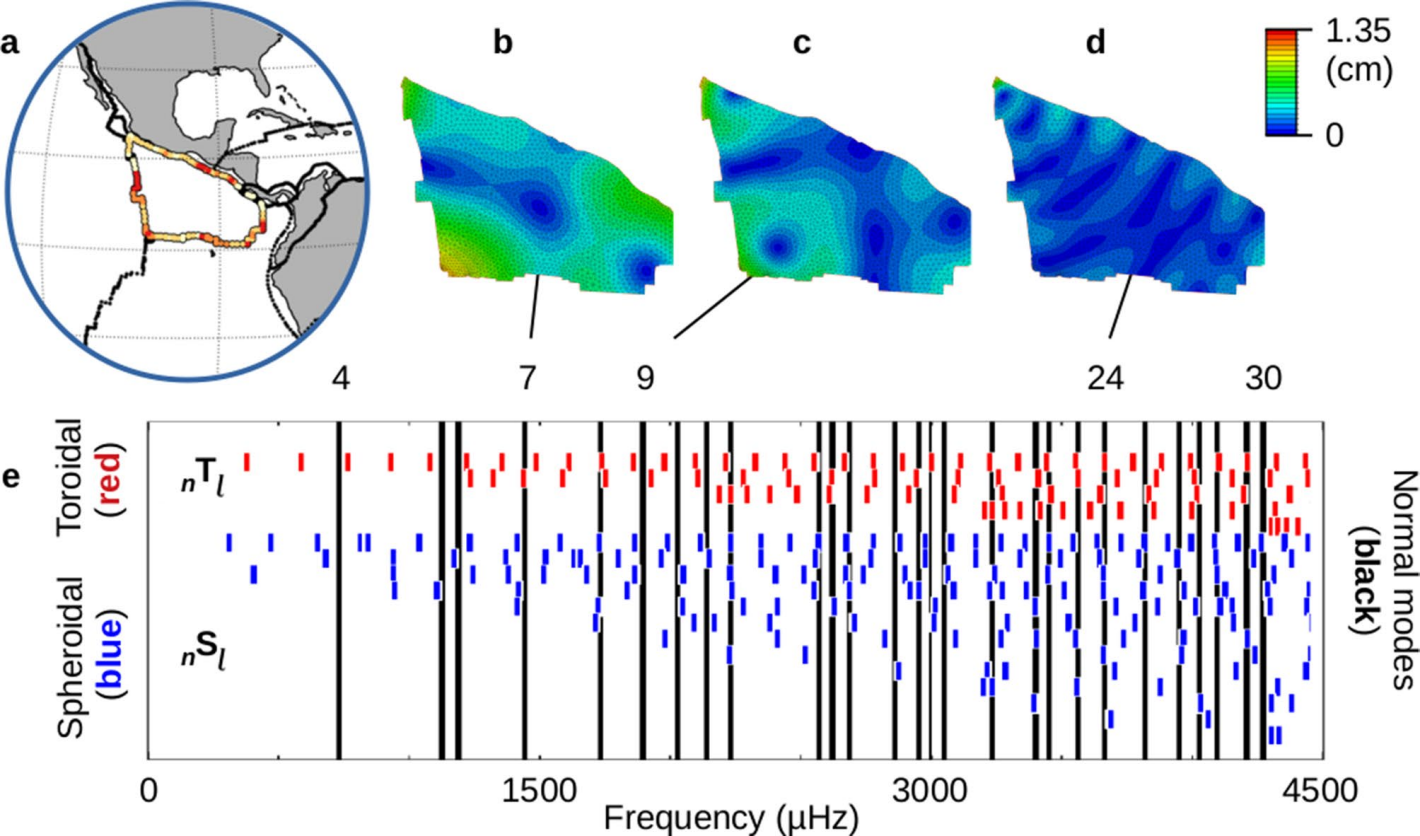
Normal Modes of the Cocos plate. (**a**) Data for the susceptibility to remote triggering extracted from Fig. 3a^12^. The most susceptible location is on the western face, with a p-value of 0.004. Other secondary highs are visible on the eastern diagonal edge and to the south. (**b**–**d**) Examples of normal modes are shown for the Cocos plate^19^. Plots are set to the same displacement scale (color bar shown). Blue represents nodal lines at the extreme. All three examples have nodal lines matching up with the main susceptibility high on the western edge along with the eastern diagonal edge. Alignment with other secondary highs is present as well. (**b**) Normal mode 7. (**c**) Normal mode 9. (**d**) Normal mode 24. (**e**) Natural frequencies are shown for normal modes 4 to 30 (black lines). Overlain are frequencies for toroidal (red) and spheroidal (blue) free oscillations^22^. The toroidal and spheroidal frequencies are organized by overtones. The top row of each represents the fundamental frequencies (_0_T_ℓ_ and _0_S_ℓ_), the next row represents the 1st overtones (_1_T_ℓ_ and _1_S_ℓ_), etc. Free oscillations of the earth are available to drive a number of the normal modes of the Cocos plate.

Our initial test is on the Cocos plate (Fig. 7a), which is somewhat triangular in shape and composed only of oceanic crust. The spacing between its susceptibility highs is about twice that of the Caribbean plate (Fig. 6a) although the areas of the two plates are quite similar. We constructed a finite element model (FEM) of the Cocos plate for input into Abaqus 6.14–1^19^. Plate boundary locations^15^ were used to define the edges of the Cocos plate, and a thickness of ~ 11 km (0.1° arc distance) was uniformly applied. For this initial model we did not add a slab to represent the subducting section diving to the northeast^20^. Density, Young’s modulus and Poisson’s ratio for oceanic crust were obtained from an FEM off the coast of Peru^21^. The bottom of the plate was pinned to allow motion only in the *ij*-plane (horizontally) and represents plate movement along the top of the lower lithosphere.

Initial results indicate examples of displacement magnitudes for normal modes for the Cocos plate with features that align with the primary susceptibility location on the western boundary, as well as other secondary highs (Fig. 7b–d). Please note that the amplitude of the normal mode displacement is not absolute, as each mode will be scaled in specific application. The frequency range of modes 4 to 30 covers 730.7 to 4270.8 μHz, or periods ranging from 22.8 to 3.9 min (Fig. 7e). These values are within the frequency range of toroidal and spheroidal free oscillations of the earth^22^ (Fig. 7e). Free oscillations (planetary standing waves) are regularly produced following large magnitude earthquakes. The source events (M ≥ M6.0) that precede the potentially triggered events (M ≥ M5.0) are large enough to generate free oscillations and can therefore stimulate specific normal modes of the plate.

We propose that specific normal modes can induce triggering (a failure along the fault plane) dependent on the associated stress. The units of stress are force per unit area, and stress is a tensor (not a vector). Positive stress normal to a plane represents bidirectional expansion, while negative stress represents compression. Here we consider what happens if we have a one-sided change in the stress field near a fault plane, but not on the other (for example, a change in the stress in the Cocos Plate but not the Pacific Plate). If we have a unit cell on one side a fault plane, then positive stress normal to the fault plane will expand into the fault plane, while negative stress represents a contraction away from the fault plane. If a one-sided contraction occurs in the stress normal to the fault plane, then the force binding the fault plane together will be reduced. Reduce the force sufficiently and a fault rupture will follow.

**Figure 8:**
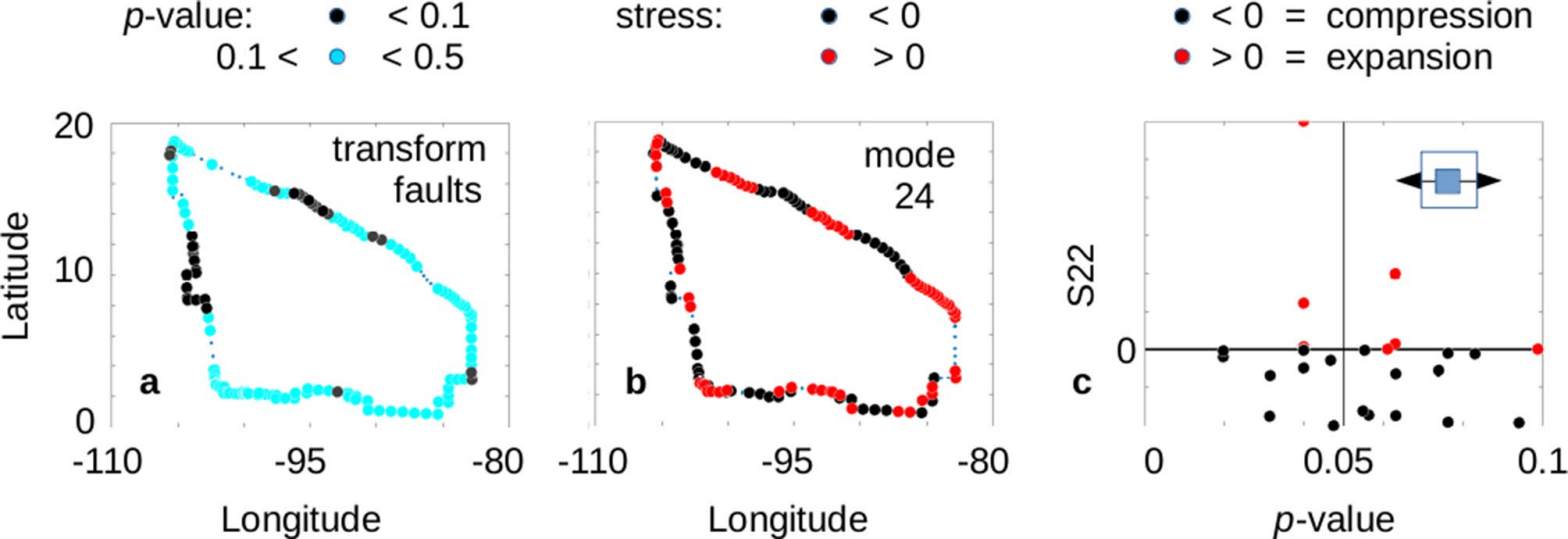
Comparison of Susceptibility to Triggering and Horizontal Stress. (**a**) Susceptibility of transform faults to remote triggering is shown in simplified form, with black circles for p-values < 0.1, and cyan dots for 0.1 < p-value < 0.5. The location of the highest susceptibility is on the western edge of the Cocos plate. (**b**) A representation of the perimeter stress due to normal mode 24 is shown. If the horizontal components (S11 and S22) are both positive, a red dot is shown; if the horizontal components are both negative, a black circle is shown. Small blue dots show those locations where S11 and S22 have opposite signs. (**c**) A scatter diagram of the highly susceptible p-values in (**a**) versus the E–W stress component (S22) of mode 24. A strong matching of negative (compressive) stress with remote triggering of transverse faulting is present.

We can do an initial test for the presence of this triggering using the data for the Cocos plate. The susceptibility high that is seen along the western edge of the Cocos Plate is due to transverse faulting. A simplified version of the transverse p-values for the Cocos Plate (Fig. 8a) shows all p-values < 0.10 as black circles and all p-values between 0.10 and 0.50 are cyan. We also have a representation of the perimeter stress values for mode 24 (Fig. 8b). Here we have compared the sign of the S11 (north–south) stress with the sign of the S22 (east–west) stress. If both are positive, we have pure horizontal expansion (red), while if both are negative we have pure horizontal compression (black). A scatter diagram of the p-values less than 0.1 versus the S22 stress (Fig. 8c) shows the majority of the susceptible locations also have negative (contracting) horizontal stress present. While some of the dots overlay each other, a tabulation shows that 22 of the 30 p-values < 0.10 are associated with negative horizontal stress. A matchup rate of 22 of 30 yields a p-value of 0.0053. Comparison of these results with ten synthetic catalogs for strike-slip events (Supplemental Information, Section 2, Fig. 8c) shows that randomized data give p-value results that range from 0.994 to 0.004, while the observed data has a higher matchup rate (smaller p-value) than 9 of 10 random catalogs. The results from the data shown in Fig. 8c are significant when compared with synthetic catalogs. The pattern in the observed data supports the idea that a temporary increase in negative horizontal stress may be involved in the triggering mechanism, while comparison with synthetic catalogs confirms this is not a random result.

## Discussion

The method developed here to detect the local expression of remotely triggered high magnitude earthquakes (Figs. 1, 2) is essentially a local test based on the retrospective analysis of *Experiment 2* of the previous study that presented high magnitude, remote triggering by global source events^8^. The resulting p-values are interpreted globally as a metric that indicates local susceptibility to remote triggering (Fig. 3). The primary conclusion of the previous paper asserting the existence of systematic triggering from anywhere beyond the aftershock zone (up to 180° away) is consistent with the results here (Supplemental Information, Section 3).

Testing for the local presence of remote triggering also yielded estimates of the globally varying onset magnitude for remote triggering (Fig. 4). Above this onset magnitude only relatively small perturbations are needed to trigger earthquakes; below this magnitude stronger forces are required. A ‘reality check’ on the results was to compare the onset magnitudes with observations of M_C_, both local studies and global analyses. While the onset magnitude was consistent with some results of local seismic studies, they in general were lower than the calculated values for the seven types of plate boundaries. We later suspected that the triggering magnitude was being lowered by a physical process that was modifying the boundary conditions along the fault planes. Given the observation of negative horizontal stress matching up with the low p-value results of transverse faults (Fig. 8), a temporary reduction in the normal forces along the fault plane could be involved, effectively reducing Mc to the observed onset magnitudes. It is noted that a linear relationship appears to be in the data between the p-value of triggering and the onset magnitude (Fig. 5) and that projecting the onset magnitudes to p-values of 1.0 gives approximate match-ups with Mc estimates. The onset magnitude progression of convergent > transverse > divergent boundaries (with subduction being the greatest) was also consistent with the progression seen in global analysis of M_C_.

Patterns of p-values around individual plates appeared to be quite distinct (Fig. 6), which led to the speculation that an external process could be modifying the boundary conditions along the edges of the plates. Such a process would also be responsible for lowering the values of M_C_ to the observed onset magnitudes. We explored one such possibility with an FEM model analyzing thin-plate resonance of the Cocos plate. More significant than the specific patterns present in the various normal modes of the Cocos plate were their natural frequencies (Fig. 7). In the case of the Cocos plate, the natural frequencies can be driven by a variety of frequencies associated with global free oscillations (Fig. 7e).

Given the possibility of normal modes of the Cocos plate being driven by specific free oscillations following source events with magnitudes ≥ M6.0, the stress tensors for normal mode 24 were examined for overall horizontal expansion or compression along the perimeter of the plate. Comparison with p-values for transform faults (0.0–0.10) showed overall association of compressive stress regimes associated with susceptibility to triggering. We suggest that the original source events (M ≥ M6.0) drive the normal mode responses of the individual plates via free oscillations, creating a mechanism for delayed triggering. As those normal modes are established, they eventually generate sufficient negative stress normal to the local fault planes, and triggered events (M ≥ M5.0) ensue.

## Conclusions

A method for analyzing a local region’s susceptibility to remote triggering is presented. While it was applied here in a uniform fashion across all plate boundaries, it can also be ‘tuned’ to a specific region. The size of the target area being tested may be smaller, change shape, or be rotated as the specific context dictates. Also obtained by this method is an empirical estimate of the onset magnitude for triggering to occur. Researchers may find this to be an appropriate proxy for a variable M_C_ in their local earthquake hazard analyses.

A three-day time period between source event and triggered event is present in the calculations, but the mechanisms implied by this delay are not specified. An intriguing possibility is that resonance of entire tectonic plates is involved, resonance driven by free oscillations created by the high magnitude source events themselves. As the individual modes are established in the hours that follow, the deviatoric stress associated with those modes builds upon the existing fault planes. Sufficient one-sided negative stress normal to the fault planes could release the triggered events.

## Methods

### Global data

Earthquake data were obtained from the United States Geological Survey (USGS) archives^23^ for all earthquakes with magnitudes ≥ M5.0. The time period covered goes from the start of 1973 through the end of 2016. A minimum threshold of M5.0 for data going back to 1979 was determined to be globally detectable in a previous study^24^. This is confirmed in the current set by examining the magnitude-frequency distribution of the data. No roll off or corner frequency is observed in the data used for this study as the magnitudes drop to M5.0. Additionally, predicted *b*-value counts are within 0% to -6% of the observed totals for magnitudes below M5.5. With no observable drop-off in the earthquake counts at the lowest magnitudes used in this study, we conclude that the global catalog is complete when using a threshold of M5.0.

These data were then compared with the results calculated by the Global Centroid-Moment-Tensor (CMT) Project^10,11^. When match-ups were available, the rake of the primary focal plane was used to classify the type of faulting. A rake of 0° ± 30°, or 180° ± 30° was defined as a strike-slip fault. A rake of − 90° ± 60° is a normal fault (where the hanging wall drops) and a rake of + 90° ± 60° is a reverse (or thrust) fault.

### Filtering out aftershocks

Care is taken to remove known clustering processes from this analysis using a standard declustering technique. The number of aftershocks follow distribution patterns based on the magnitude of the main event, such as the ‘epidemic type’ model^25^. Our concern here is to decluster the data over time and remove the effects of this known triggering process.

The declustering method selected was the windowing technique applied by Gardner and Knopoff^9^. While this method is easy to implement, importantly the temporal distribution of the earthquakes in the declustered catalog follows a Poisson distribution^9,26^. Using this method, an earthquake has both a spatial and a temporal extent which are functions of magnitude. The equations used are given in Eq. (1) of van Stiphout et al.^26^. Specifically, the distance window (in kilometers) is estimated by 10^0.1238M+0.983^. The time window (in days) has one curve for M6.5 and larger (10^0.032M+2.7389^) and another for smaller magnitudes (10^0.5409M-0.547^). The temporal extent can be quite significant, as data from large magnitude events extend hundreds of days. For example, the time window for M5.0 covers 144 days, M6.0 goes 499 days, M7.0 is at 918 days, and M8.0 extends to 988 days. Similarly, the distance window for M5.0 covers 40 km, M6.0 is at 53 km, M7.0 spans 71 km, and M8.0 extends to 94 km. Data that fall in the combined spatial and temporal windows, either before or after a main event, are flagged as aftershocks or foreshocks and removed from the subsequent analysis.

### Data selection for a project area

Given a latitude and longitude (lat0 and lon0) for a location of interest, the global data (filtered for aftershocks) are searched to locate all earthquakes within a box defined by a minimum and maximum latitude and longitude. The bounding box in this study is defined by lat0 + /− dlat and lon0 +/− dlon, where dlat is set at 2.85° latitude and dlon is set at 2.85/sin(90-lat0) degrees longitude.

Once a bounding box is defined, the global data set (with aftershocks removed) is searched for any earthquakes with magnitudes M5.0 or larger within the project area. This is the set of potentially triggered events. The global data set is then searched again for any earthquakes of magnitude M6.0 or larger from anywhere on the globe that took place within 72 h of the potentially triggered events. These large, global earthquakes are the source events. We now have ‘n’ potentially triggered events, and ‘x’ of those are preceded by one or more source events within 72 h. An analysis of the global set used in this study shows that the probability of observing one or more source events within 72 h of a potentially triggered event has a probability of 0.501. This value is applied to all regions.

### Calculation of p-values

The general binomial distribution is of the form

2 $${\text{f}}\left( x \right) = \left[ {n!/\left( {n - x} \right)!} \right] \, *p^x * \, \left( {{1} - p} \right)^{\left( {n - x} \right)}$$

The first part (with factorials) is generally read “*n* choose *x*” where ‘*x*’ is the number of successes out of ‘*n*’ trials, and the probability ‘*p*’ gives the probability of a success.

Each project area supplies ‘n’ earthquake counts of potentially triggered events. The number of triggered events with observed source events in the preceding 72 h plays the role of ‘*x*’ in the binomial distribution. The random probability of having such a ‘success’ for the global set used in this study was found to be 0.501.

What is now needed is a cumulative probability function for the binomial distribution which will give the total probability of getting up to ‘*x*’ successes. In R^27^, this is given by the function ‘pbinom’. If *cpf* = pbinum(*count*, *size*, *prob*), then the p-value is given by 1 − *cpf*. The p-value thus gives the probability that the random probability 0.501 could deliver a count of ‘*x*’ or more.

The last detail is that we calculate the mid p-value^28^ using [*count* − 0.5] instead of the traditional p-value, to avoid the known bias appearing in the binomial calculation based on discrete quantity that take only integer numbers (such as earthquake counts).

### Algorithm for analysis

We wish to evaluate the p-values as a function of magnitude for a given project area. The p-value will then be associated with earthquakes within a magnitude range that runs from a lower bound to an upper bound. The upper bound is set at the maximum magnitude event within the project area. The lower bound is iteratively adjusted to cover the range from M5.0 to the upper bound magnitude. The results yield a p-value curve which is a function of the lower bound magnitude, running from M5.0 to the maximum observed earthquake in the area.

Given the p-value curve running from M5.0 to the maximum magnitude event, it is now searched for the magnitude with the minimum p-value. In general, this is visually easy to locate if one plots a -log10 transform of the p-value curve. The onset magnitude for triggering becomes the magnitude with the minimum p-value for this project area. The minimum p-value indicates the susceptibility to remote triggering within the bounding box.

### Onset magnitude detail

The simplest version of this algorithm locates the magnitude associated with the actual minimum p-value and assigns that magnitude as the onset magnitude for remote triggering. For p-values smaller than 0.05, however, it was of interest to see how low the magnitude would go while still allowing the overall p-value to still be less than 0.05 (highly susceptible to triggering). The onset magnitude in this case indicates an overall p-value zone that is less than 0.05. An additional adjustment for this particular situation is to make sure the ratio of source events to total events for the onset magnitude bin is greater than the general probability of 0.501. If it isn’t, the onset magnitude is increased by one bin and tested again (each bin spans one tenth of a magnitude). When done in this fashion, the algorithm picks onset magnitudes consistent with those found by visual inspection of the data.

## Supplementary Information

Supplementary Information.
