# Supplementary material for: Remote triggering of high magnitude earthquakes along plate boundaries
Source: Sci Rep. 2022 Jan 21;12:1138. doi: 10.1038/s41598-022-05102-4 (PMC8782900; doi:10.1038/s41598-022-05102-4)
Supplement: Supplementary file 1 — Supplementary Information. [file 41598_2022_5102_MOESM1_ESM.pdf]

Supplementary Information for:

REMOTE TRIGGERING OF HIGH MAGNITUDE EARTHQUAKES ALONG PLATE BOUNDARIES

Robert T. O'Malley[1*], Ayush Choudhury[2] & Yue Zhang[2]

---

1 Department of Botany of Plant Pathology, Cordley Hall 2082, Oregon State University, Corvallis, OR 97331-2901, USA (email) omalleyr@science.oregonstate.edu (phone: 541-737-2316)

2 School of Electrical Engineering and Computer Science, 3117 Kelley Engineering Center, Oregon State University, Corvallis, OR 97331

November 04, 2021

* To whom correspondence should be addressed

Section 1: prob = 0.501

In this study we examined the declustered earthquake catalog to find how often potentially triggered events (M ≥ M5.0) are preceded within three days by one or more potential source events (M ≥ M6.0). This ratio was almost exactly equal to 50% (prob = 0.5010).

If we consider taking subsets of the declustered earthquake catalog for normal, strike-slip and reverse events (based on CMT matchups) we obtain very similar results: normal = 0.4942, strike-slip = 0.5067, and reverse = 0.5013.

If we construct multiple synthetic (time-randomized) earthquake catalogs, we get the following results: prob = {0.5090, 0.5020, 0.5021, 0.5068, 0.5060, 0.4996, 0.5021, 0.5108, 0.5061, 0.5055}. This random set has an average of 0.5050 and a standard deviation of 0.0035. This is slightly higher (0.004) than the average value used in the study.

The time series of the change in the value of ‘prob’ is also interesting (Figure S1). The rise in the ‘prob’ value over time is likely linked to the observed increase in high magnitude earthquakes over time. However, the increase was not viewed as statistically significant [***ref 1***] implying that the global average is appropriate over the time span (ie, a single stationary process, rather than shifting from one to another).

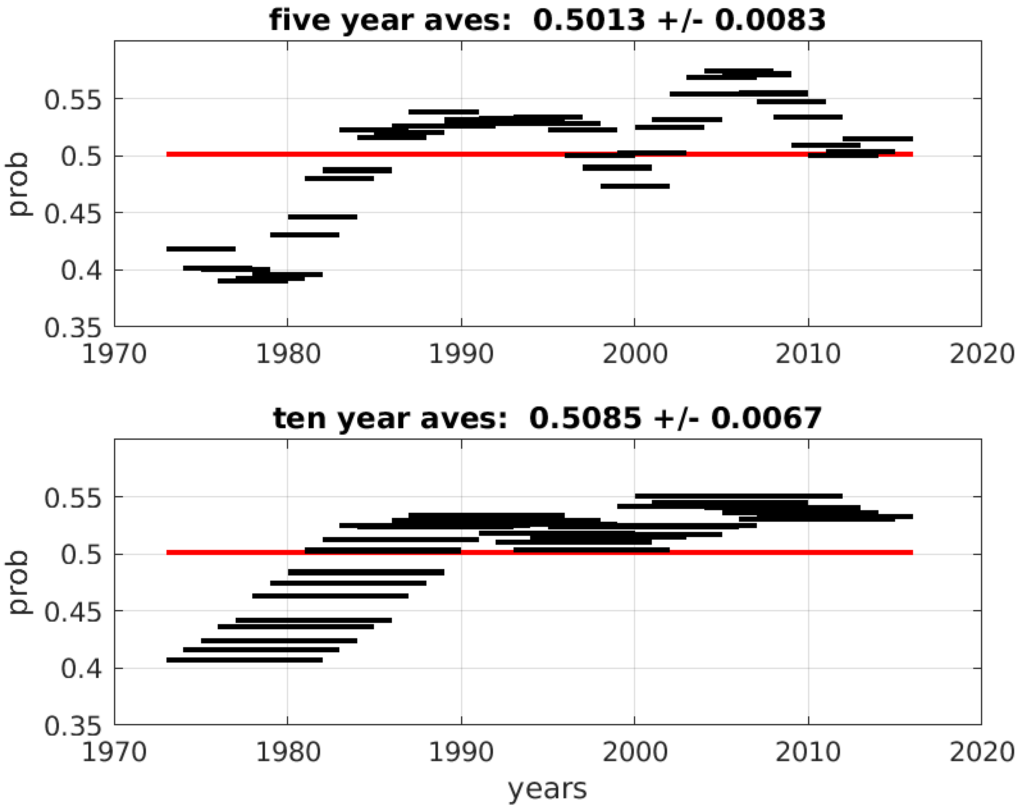

Figure S1: Time series of ‘prob’ over 44 years.

The red horizontal line indicates the average value used over the time of the study (‘prob’ = 0.501); the black horizontal lines indicate the time span for each estimate. The average of these estimates is indicated above each panel, while the +/- value is one standard error, indicating the range that is possible for the average value. The top panel is for running 5-year averages; the bottom panel is for running 10-year averages.

Section 2: Comparisons of results with synthetic (randomized) earthquake catalogs

It can be common to have patterns asserted which are not statistically significant, particularly in the field of earthquake triggering [***ref 2, ref 3***]. While the presence of delayed, high magnitude triggering by source events has been shown to have a strong statistical argument [***ref 4***], it is still interesting to compare the results obtained in this paper from real data against those based on synthetic (time-randomized) earthquake data. Synthetic catalogs were therefore constructed by assigning randomized days and time-of-day to all earthquakes used in this study. Any day within the 44 years of the study was equally possible. These synthetic sets were used in an attempt to reproduce the significant details shown in the figures of the paper. The ability of the synthetic sets to reproduce these results are discussed in the following subsections.

- Figure 1a results

Figure 1a of the text shows an example where 23 of 25 of the highest magnitude thrust earthquakes are preceded by source events within three days. The *p*-value of this being a result of a random occurrence is found to be 4.5 e-05 (*p*-value = 0.000045). Using ten synthetic catalogs for thrust events, the number of source events found for the top 25 magnitude events are {11, 13, 15, 10, 15, 16, 13, 12, 13, 12}. This leads to associated p-values of {0.7244, 0.4260, 0.1657, 0.8387, 0.1657, 0.0857, 0.4260, 0.5810, 0.4260, 0.5810}. Ten out of ten of the p-values are greater than the observed result of 0.000045.

If one generalizes the problem (instead of requiring the extraction of the top 25 magnitude thrust events) we can calculate the minimum p-values generated by the synthetic sets. This results in the following: {0.2505, 0.1255, 0.0355, 0.1538, 0.0630, 0.0251, 0.1255, 0.2392, 0.1456, 0.3135}. All ten synthetic catalogs resulted in *p*-values greater than the observed result, and in eight out of ten times, these were based on no more than the top 7 magnitude thrust events.

- Figure 2a results

Similar to Figure 1a of the text, Figure 2a is based on results for reverse faulting. In this example 19 of 23 of the largest reverse events were preceded by source events. The *p*-value of this being a result of a random occurrence is found to be 7.94 e-04 (*p*-value = 0.000794). Using ten synthetic catalogs for reverse events, the number of source events found for the top 23 magnitude events are {13, 15, 13, 9, 13, 13, 12, 11, 12, 12}. This leads to associated p-values of {0.2735, 0.0770, 0.2735, 0.8483, 0.7735, 0.2735, 0.4227, 0.5839, 0.4227, 0.4227}. Ten out of ten of the *p*-values are greater than the observed result of 0.000794.

If one generalizes the problem (instead of requiring the extraction of the top 23 magnitude reverse events) we can calculate the minimum *p*-values generated by the synthetic sets. This results in the following: {0.2735, 0.0315, 0.0199, 0.2505, 0.1504, 0.0908, 0.1345, 0.1884, 0.0356, 0.4149}. All ten synthetic catalogs again resulted in *p*-values greater than the observed result. In this case, two of the three smallest *p*-values were again calculated based on 7 or fewer of the top magnitude reverse events.

- Figure 2c results

Figure 2c is based on results for transverse faulting. In this example 14 of 17 of the largest transverse events were preceded by source events. The *p*-value of this being a result of a random occurrence is found to be 0.0038. Using ten synthetic catalogs for transverse events, the number of source events found for the top 17 magnitude events are {10, 4, 9, 8, 11, 10, 11, 5, 9, 10}. This leads to associated p-values of {0.2426, 0.9848, 0.4101, 0.5956, 0.1204, 0.2426, 0.1204, 0.9526, 0.4101, 0.2426}. Ten out of ten of the *p*-values are greater than the observed result of 0.0038.

If one generalizes the problem (instead of requiring the extraction of the top 17 magnitude reverse events) we can calculate the minimum *p*-values generated by the synthetic sets. This results in

the following: {0.0628, 0., 0.5009, 0.2644, 0.5609, 0.0693, 0.0222, 0.1204, 0.7505, 0.2505, 0.1101}. All ten synthetic catalogs again resulted in *p*-values greater than the observed result, and in six out of ten cases these results were based on no more than the top 6 magnitude thrust events.

- Figure 2b results

Figure 2b of the text shows the results for normal events, and yields a result indicating that triggering is not happening. The *p*-value shown here is 0.6895, which is the lowest the *p*-value gets, and this value is based on only the top 3 magnitude normal events. No triggering is implied from the beginning, and triggering is never suggested as the magnitude range extends to lower magnitudes.

Because the number of samples is so low (only the top three magnitude events) comparison with random selections will not be unique. With only 4 possible outcomes for the possible number of observations with source events, all were observed in the ten random tests. A different metric is required to differentiate the observed results from the synthetic sets.

The *p*-value results for Figure 2b comes very close to approximating a straight line with a negative slope. It indicates that the *p*-values were always greater than 0.5 at the highest magnitudes, and they get even larger as more and more samples are included as we sample lower magnitudes. If we fit a best-fit linear curve to the result, and calculate the average absolute value misfit for all bins, we get a linear misfit of 0.0351 for the data. Doing the same for ten synthetic sets of normal earthquakes gives the following average absolute value misfits for all bins: {0.0989, 0.0708, 0.0765, 0.0805, 0.1070, 0.1025, 0.1932, 0.1358, 0.1122, 0.1070}. These misfits for synthetic data all range from 2x to 6x that of the observed data. In general, this indicates a greater coherence across magnitude ranges for the real data than that found in random sets.

- Figure 3b

When examining the maps shown in Figure 3 in the text, the strike-slip *p*-values (Figure 3b) show a greater amount of "red" (*p*-values < 0.05) than either of the maps for reverse or normal events. We would like to test this prevalence of low *p*-values (Figure S2) with those found in time-randomized sets of global strike-slip earthquakes (Figure S3, S4). The examples shown in Figures S3 and S4 are from two of the ten synthetic strike-slip catalogs.

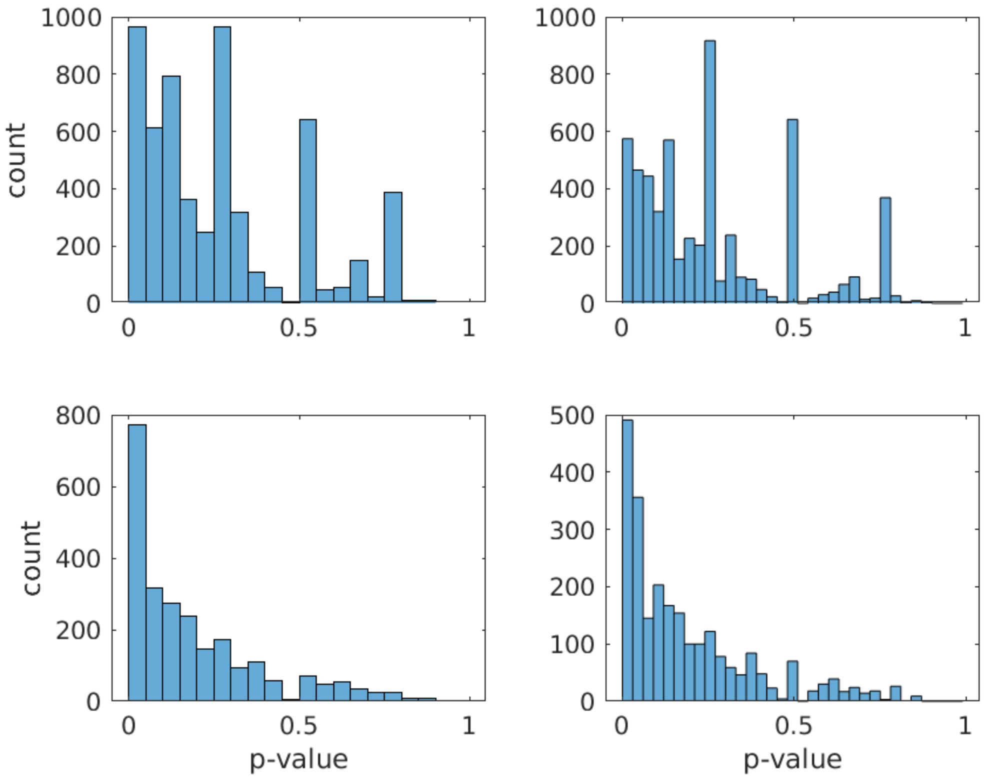

Figure S2: Global histograms of *p*-values for observed strike-slip events (Figure 3b, text)

The upper-left panel is for all results, using a bin-size of 0.05. The spikes seen in counts are a result of *p*-value analyses run on small sample sizes. For example, the lower-left panel is restricted to those results with seven or more high magnitude earthquakes. The panels on the right side offer similar results while using a smaller (0.03) bin size.

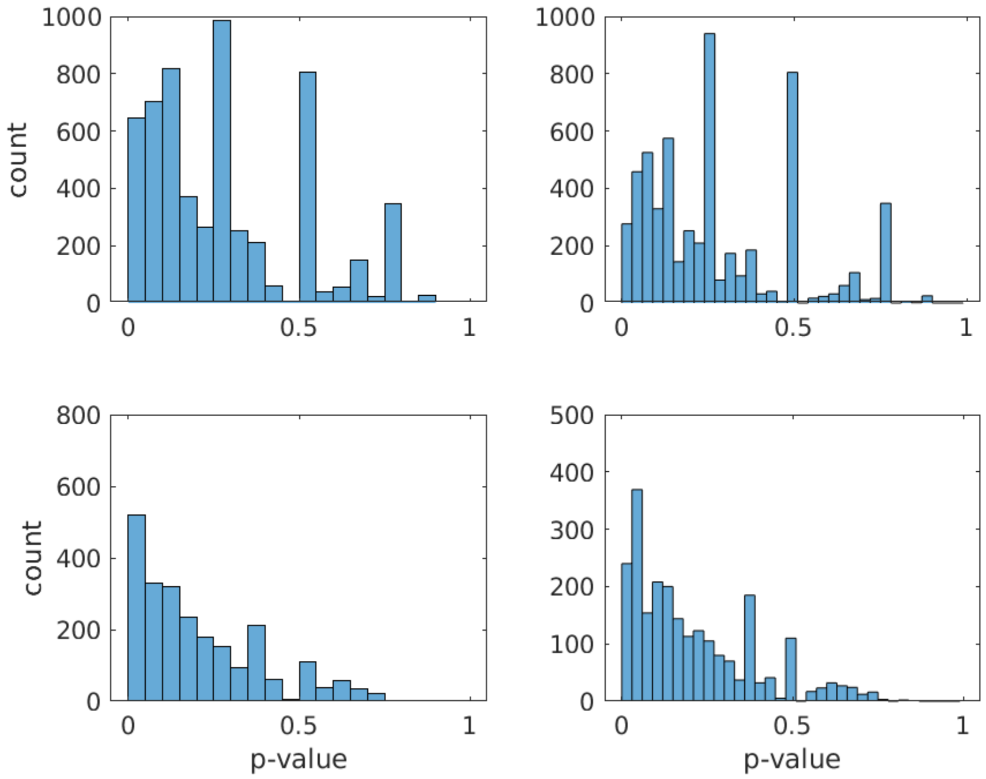

Figure S3: Example 1 - global histograms of *p*-values for synthetic strike-slip events

The upper-left panel is for all results, using a bin-size of 0.05. The lower-left panel is restricted to those results with seven or more high magnitude earthquakes, while using a 0.05 bin size. The panels on the right side offer similar results while using a smaller (0.03) bin size.

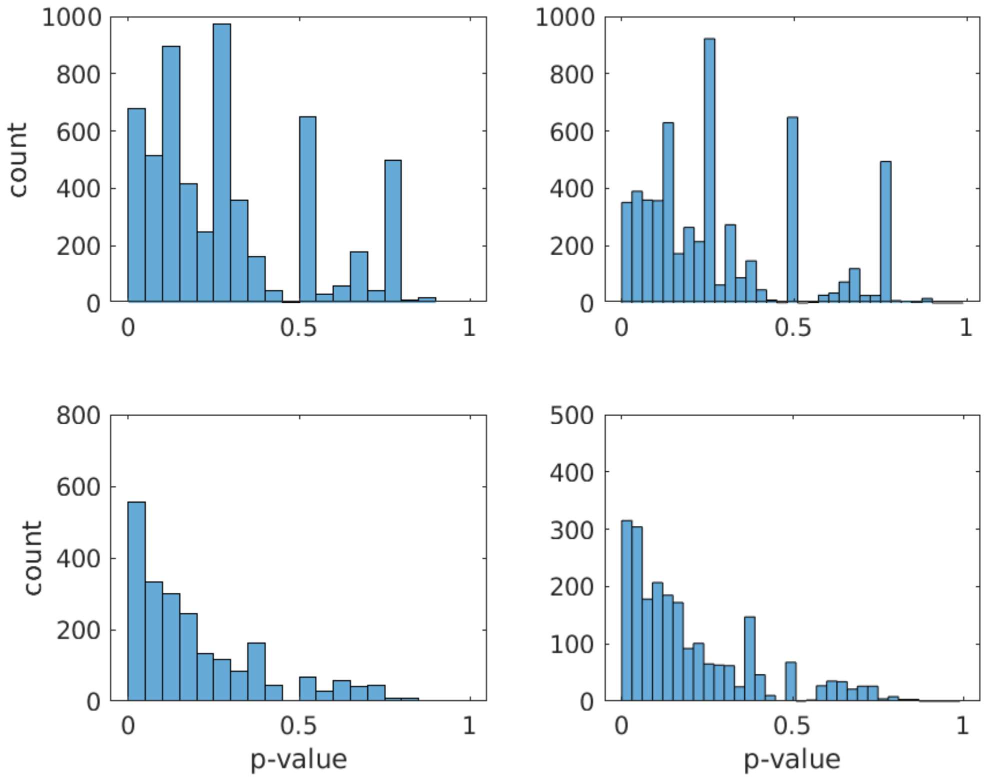

Figure S4: Example 2 - global histograms of *p*-values for synthetic strike-slip events

The upper-left panel is for all results, using a bin-size of 0.05. The lower-left panel is restricted to those results with seven or more high magnitude earthquakes, while using a 0.05 bin size. The panels on the right side offer similar results while using a smaller (0.03) bin size.

These data appear to be log-normally distributed. In order to test the prevalence of the lowest *p*-values in the strike-slip data, we will compare the count of the first bin shown in the data with that found in the ten synthetic datasests. We will test two cases: *p*-value < 0.05 and *p*-value < 0.03 (Table S1).

Table S1: Global tally of low *p*-values for strike-slip events

Data source	Count for p-value < 0.05	Count for p-value < 0.03
Baseline (observed data)	975	575
Synthetic set		
01	646	276
02	621	278
03	482	227
04	708	411
05	638	233
06	853	398
07	593	269
08	677	351
09	840	432
10	874	431
Mean value	693.2	330.6
Standard deviation	127.1	82.7
Baseline	+2.1 sigma	+2.9 sigma

The low *p*-value tallies from the strike-slip global map (shown in Figure 3b of the text) are clearly anomalous compared to the ten synthetic data sets. The count is higher than 10 out of 10 of the randomized sets, and the baseline level exceeds 2 sigma. The case is the most evident when examining the smaller bin size of values < 0.03, where the baseline is +2.9 sigma above the mean.

- Figure 3c

Doing the same type of analysis for the reverse (thrust) events does not result in such a striking result. For *p*-values < 0.05, the baseline count is greater than 8 of the 10 random sets, but is only +0.40 sigma above the mean. A stronger result is present for *p*-values < 0.03, where the baseline is greater than 9 of

the 10 random sets, and the baseline is now +0.96 sigma above the mean. Strong support for the uniqueness of the reverse events will be found in the evaluation of Figure 5 results (see below).

- Figure 3d

The global *p*-values in the text for normal earthquake (Figure 3d) show a greater amount of "green" (*p*-value > 0.50) than either of the maps for strike-slip or reverse events. Here we would like to test this prevalence of observed large *p*-values (0.5 < *p*-value < 0.7) with those found in time-randomized sets of global normal earthquakes. Similar to Figure 2b, this is testing on anomalous levels of 'no-triggering' for normal events.

The results (Table S2) indicate that the baseline count for the 0.5 < *p*-value < 0.7 is greater than the randomized set counts ten out of ten times. Moreover, the baseline count is +2.3 sigma above the mean of the synthetic sets.

- Figure 4b

Initial inspection of the global histograms for the onset magnitudes for strike-slip earthquakes (Figure 4b, text) suggested that the baseline data had fewer total events starting above magnitude 6 than the randomized sets. We examined three different bin tallies: M > 6.0, M > 6.1, and M > 6.2. The baseline tallies were less than the randomized sets eight of ten times, nine of ten times and nine of ten times respectively. The baseline moves from -0.71 sigma below the mean, to -1.2 sigma, to -1.4 sigma. The combination of the baseline tally being less than the synthetic nine times out of ten, and sitting below the synthetic mean by -1.2 sigma is suggestive, but not overwhelming.

Strong support for the uniqueness of the various onset magnitude estimates will be found in the evaluation of Figure 5 results (see below).

Table S2: Global tally of high *p*-values for normal events

Data source	Count for 0.50 < *p*-value < 0.70
Baseline (observed data)	310
Synthetic set	
01	267
02	213
03	189
04	281
05	243
06	178
07	243
08	209
09	266
10	208
Mean value	229.7
Standard deviation	35.3
Baseline	+2.3 sigma

- Figure 5

Figure 5a and 5b of the text offer an excellent opportunity to compare patterns based on extracted data (from Figures 3 and 4 in the text) against those of synthetic sets. Global data are extracted for all three different types of earthquakes and analyzed for all seven types of tectonic boundaries. The data are binned based on their *p*-value range (0.0-0.1, 0.1-0.2, and 0.2-0.3) for each tectonic regime. Average values for onset magnitudes are determined for each bin. These three averages are then used to fit a straight line for each tectonic regime and magnitudes are projected to a *p*-value = 1.0. It is possible to test the projected magnitudes for all seven tectonic types against the results obtained from synthetic

catalogs (Table S3). Italic numbers indicate magnitudes less than the observed result (shown at the top of the column), while bold numbers indicate magnitudes which are greater.

Table S3: Projected Onset Magnitudes for all Tectonic Regimes

	Subduction	Convergent		Transverse		Divergent	
**Data source**		Continental	Oceanic	Continental	Oceanic	Continental	Oceanic
Baseline (observed)	9.3731	9.0197	7.9360	7.9317	7.0503	6.4848	6.3032
Synthetic							
01	*8.5399*	*8.1545*	*7.2306*	*7.0557*	*6.6533*	*6.2020*	**6.5820**
02	*8.5719*	*7.9412*	*7.6171*	*7.1751*	*7.0135*	**6.7983**	**6.3059**
03	*7.7179*	*7.8668*	*7.2317*	**8.0154**	**7.2220**	**6.8476**	**6.6123**
04	*7.9106*	*7.4002*	*6.4756*	*7.0103*	*6.9959*	**7.8486**	**7.1893**
05	*7.1213*	*7.1015*	*6.3738*	*6.5081*	*6.4868*	**6.8453**	**6.4805**
06	*6.4453*	*6.7071*	*6.0524*	*7.3388*	**7.0710**	**7.2420**	**6.9740**
07	*6.9179*	*6.5835*	*5.8840*	*7.4657*	*6.9423*	**6.6166**	**6.4543**
08	*8.3984*	*8.0159*	*7.9112*	*7.0870*	**7.2032**	**6,4872**	*6.1795*
09	*7.8781*	*7.3771*	*6.8118*	*6.9491*	*6.3370*	**7.3347**	**6.6600**
10	*8.1251*	*7.8952*	*7.5853*	*6.9676*	*6.8621*	**6.6959**	**6.5617**
Mean value	7.7626	7.5043	6.9174	7.1427	6.8787	6.8918	6.5949
Std dev	0.7220	0.5610	0.7022	0.3778	0.2971	0.4715	0.3047
Baseline	+2.23 sdev	+2.70 sdev	+1.45 sdev	+2.09 sdev	+0.58 sdev	-0.86 sdev	-0.96 sdev
# > ratio	10:10	10:10	10:10	9:10	7:10	1:10	1:10

If there were no distinction between the real data and the synthetic, the synthetic results would be scattered around the observed baseline. This is not the case. All baseline observations of convergent data (subduction zones, continental convergence and oceanic convergence) are clearly anomalous to the randomized sets, where 30 out of 30 projected magnitude estimates are greater than the synthetic

results. The transverse results are anomalous for continental strike-slip zones (nine of ten estimates greater than synthetic data, and just over two sigma above their mean), while the oceanic transverse is not conclusive on its own (7:10 and +0.58 sigma). The normal earthquakes are also anomalous, but these data are all globally low (18 of 20 results have the observed data lower than the synthetic results) and both are on the order of 0.9 sigma below the mean. If we exclude the oceanic transverse case, anomalous results for real data were found in 57 out of 60 comparisons with random sets. That yields a *p*-value of 1.5e-15, a remarkably low value indicating the chances that 57 out of 60 could result from synthetic data. Including the oceanic transverse case, we have 64 out of 70 (p-value = 1.1e-14).

- Figure 6

Figure 6 in the paper shows *p*-value patterns around six different tectonic plates. Without an external criterion, it is difficult to construct a metric to show that these patterns are anomalous from those patterns formed by synthetic sets. All that is assured is that the patterns will be different.

Consider how well observed data with *p*-value < 0.05 (from Figure 3a of the text) compare with two examples of synthetic data (Figure S5 and S6). In both examples, less than 1/3 of the low *p*-value data pair up with equally low *p*-values from synthetic set (example 1 = 0.27 of the data are paired together, and example 2 = 0.32 matchup rate). This ratio will decrease somewhat if we examine the normal, strike-slip and thrust components of the catalog individually. While those data may vary between 0.25 and 0.30 , in general one can say two out of three of the red areas will likely not be present in the same locations in the randomized extractions. What is needed is a comparison with an external criterion for pattern matching, and analysis of Figure 8 will give us that chance (see below).

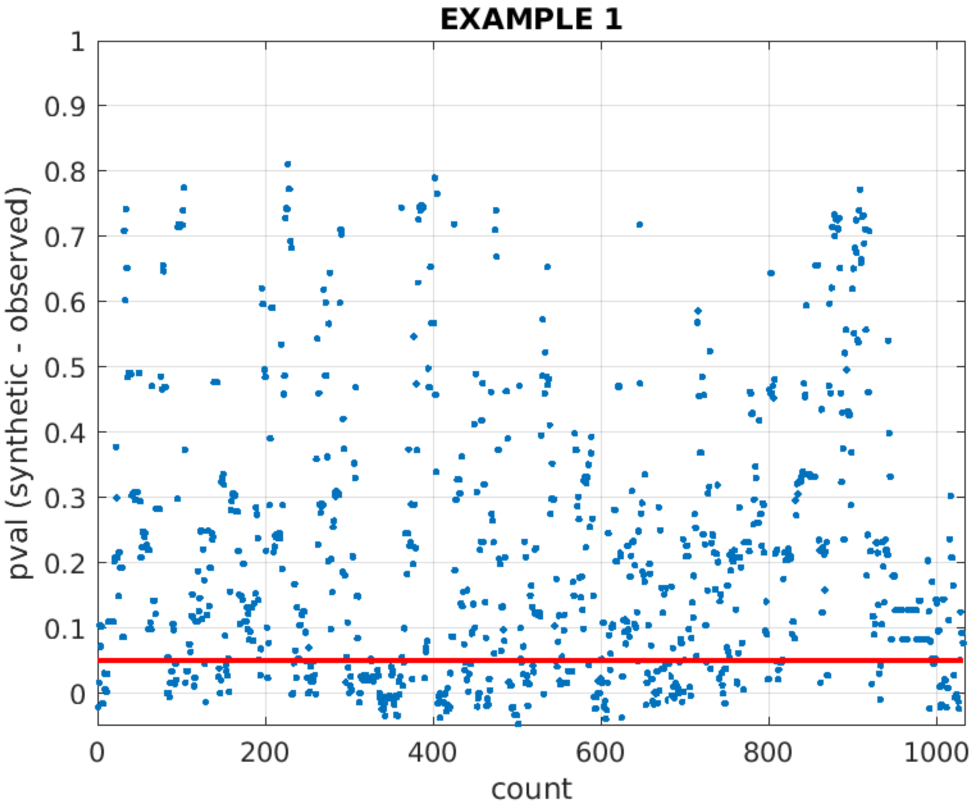

Figure S5: Example 1 – scatter plot of *p*-values for synthetic aggregate events

A total of 1032 samples were extracted from Figure 3a (text) with *p*-values < 0.05. Matching those samples up with a synthetic version of the aggregate catalog yielded 277 synthetic results that also had *p*-values < 0.05. That leads to a ratio of 0.27 matches for red areas shown in Figure 3a and Figure 6 in the text.

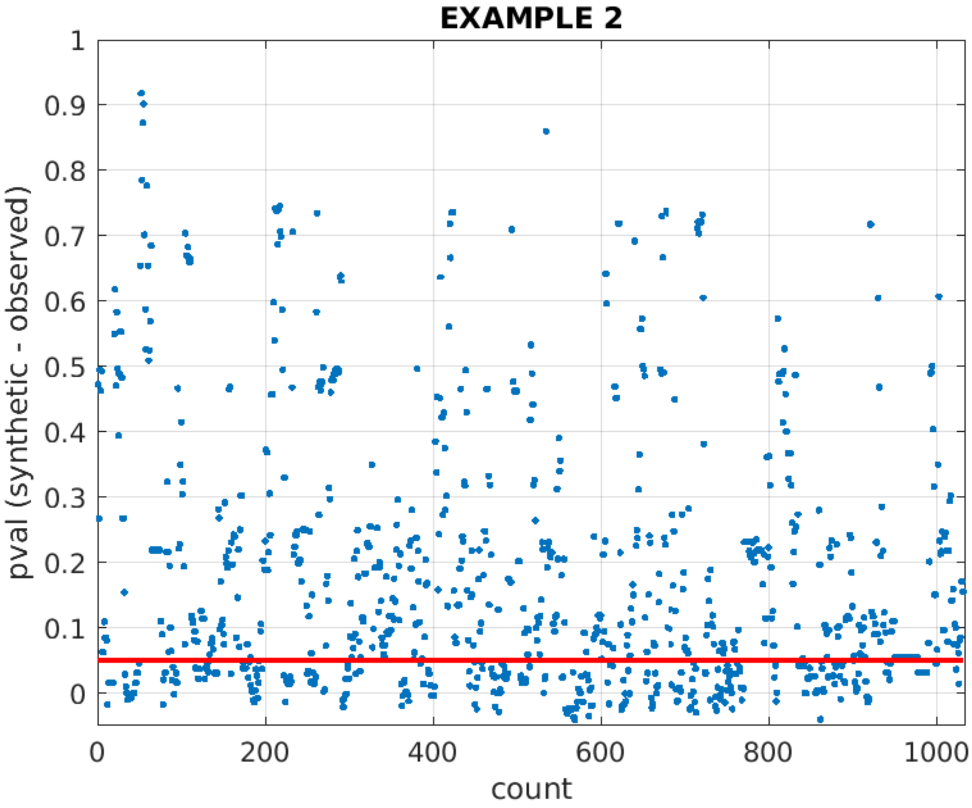

Figure S6: Example 2 – scatter plot of *p*-value matches for synthetic aggregate events

A total of 1032 samples were extracted from Figure 3a (text) with *p*-values < 0.05. Matching those samples up with a second synthetic version of the aggregate catalog yielded 338 synthetic results that also had *p*-values < 0.05. That leads to a ratio of 0.32 matches for red areas shown in Figure 3a and Figure 6 in the text.

- Figure 8c

Figure 8a (text) shows the *p*-values around the Cocos plate based on transform faults. Those locations with *p*-values < 0.1 are indicated in black on the plot. Figure 8b shows the boundary conditions for the horizontal stress of the Cocos plate based on normal mode 24 for the plate. Figure 8c shows the extent of matches between the *p*-values < 0.1 and the locations of negative horizontal stress. The horizontal stress was represented using S22 to simplify the analysis.

In 22 out of 30 cases, low *p*-value samples were matched with the locations of negative horizontal stress. This comparison with an external criterion makes for an excellent opportunity to compare the results with synthetic data. See Table S4. Italic numbers indicate synthetic *p*-values less than the observed result, while bold numbers indicate *p*-values which are greater.

Table S4: P-value matches with negative horizontal stress from mode 24

**Data source**	Negative S11 matches	Total count	p-value
Baseline (observed)	22	30	0.0053
Synthetic			
01	32	55	**0.1144**
02	20	35	**0.2025**
03	8	21	**0.8596**
04	12	29	**0.8192**
05	19	34	**0.2498**
06	25	71	**0.9936**
07	22	32	**0.0173**
08	27	44	**0.0676**
09	21	28	*0.0041*
10	35	63	**0.1909**
Mean value			0.3516
Std dev			0.3822

The synthetic *p*-values which match with the negative horizontal stress field of mode 24 are broadly distributed from 0.994 to 0.004. The large standard deviation quantifies what we see by looking at the range of values going from zero to one. The *p*-value of the observed data is smaller than 9 of the 10 synthetic results, and is anomalous when compared with the synthetic data. Being anomalous 9 of 10 times yields a *p*-value of 0.006, which indicates the chances that the observed baseline value could be the a result of the synthetic data.

Section 3: Examples of global distribution of source events

It may be of interest to some readers to see the global distribution of source events for the examples shown in the text. All maps are made with MATLAB [**ref 5**]. In this section we expand on the results for Figures 1a, 2a and 2c from the text. Even though these are from a limited set of samples, the distribution of source events can be compared favorably with Figure 4 of O'Malley et al, 2018 [***ref 4***].

- Figure 1a

Figure 1 shows an example for a portion of the subduction zone off Peru. A total of 23 of the top 25 magnitude thrust events were all preceded by one or more source events within 72 hours. Figure S7 shows the source events on a global map (red circles) along with histograms showing the counts vs arc-distance. One version of the histogram uses 15-degree binning (lower left panel) while the other shows finer resolution with 5-degree binning (lower right panel) The upper right panel of Figure S7 shows the target area to see the location of nearby source events. The one source event within the project area is more than 100 km away from its potentially triggered event, and the aftershock / foreshock algorithm found them to be independent events.

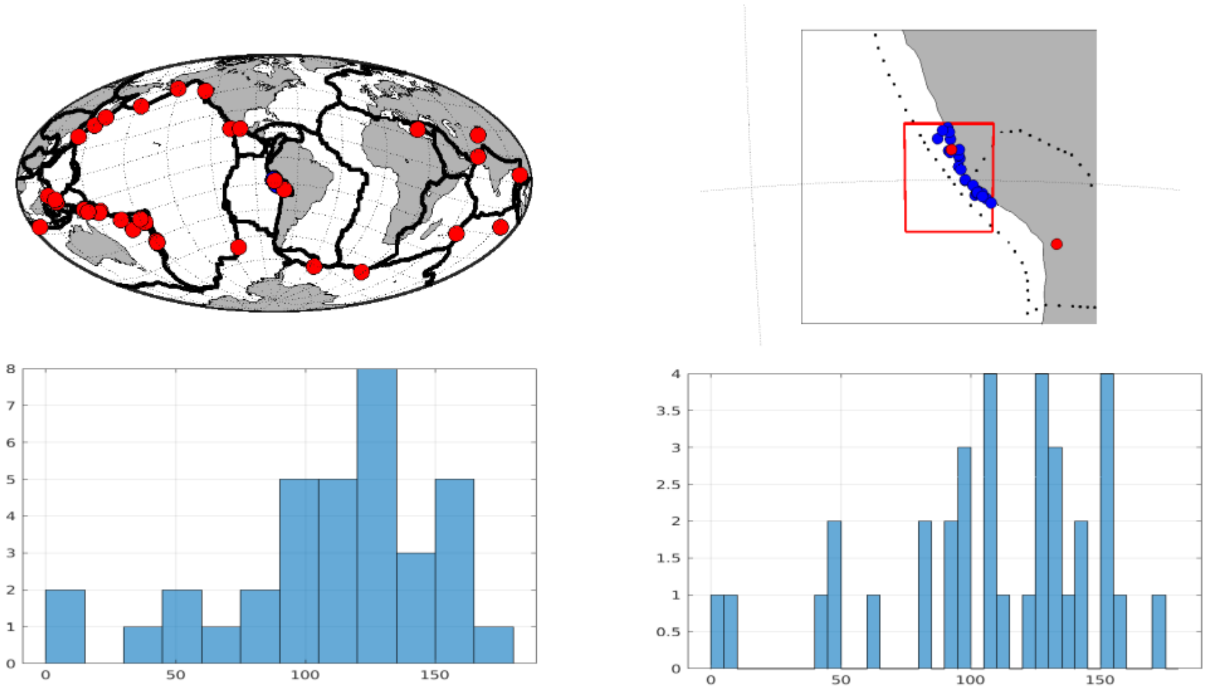

Figure S7: Source event distribution for Figure 1a (text)

Figure 1a indicates 23 potentially triggered events. Those 23 events were all preceded by one or more source events, resulting in 35 source events. Locations are shown in the top panels, with a global map on the left and the study area on the right (source events are in red and triggered events are in blue). Spatial distributions are shown in the bottom panels (15-degree binning on the left, and 5-degree binning on the right).

- Figure 2a

Figure 2a indicates triggering for thrust events with 19 of the top 23 magnitude events preceded by source events. We show global and local locations in the upper panels (Figure S8) along with 15-degree and 5-degree binned histograms of the spatial distributions in the lower panels.

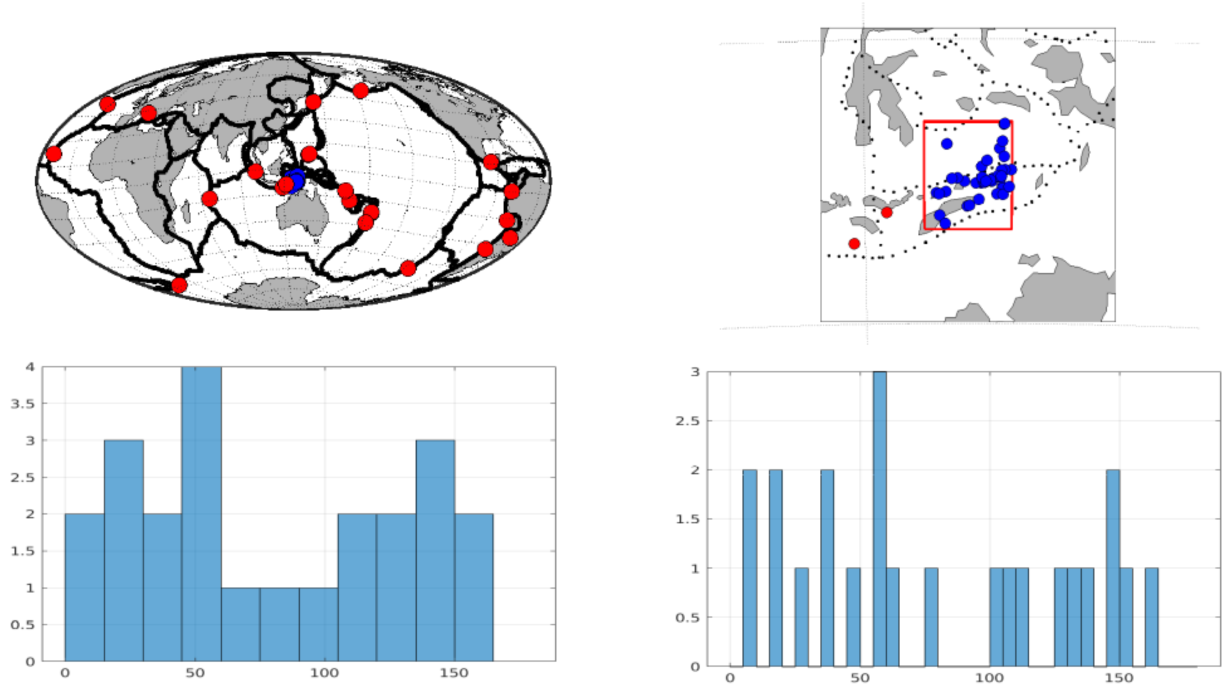

Figure S8: Source event distribution for Figure 2a (text)

Figure 2a indicates 19 potentially triggered events. Those 19 events were all preceded by one or more source events. Locations are shown in the top panels, with a global map on the left and the study area on the right (source events are in red and triggered events are in blue). Spatial distributions are shown in the bottom panels (15-degree binning on the left, and 5-degree binning on the right).

- Figure 2c

Figure 2c indicates triggering for strike-slip events, with 14 of the top 17 magnitude events preceded by source events. We again show global and local locations in the upper panels (Figure S9) along with 15-degree and 5-degree binned histograms of the spatial distributions in the lower panels.

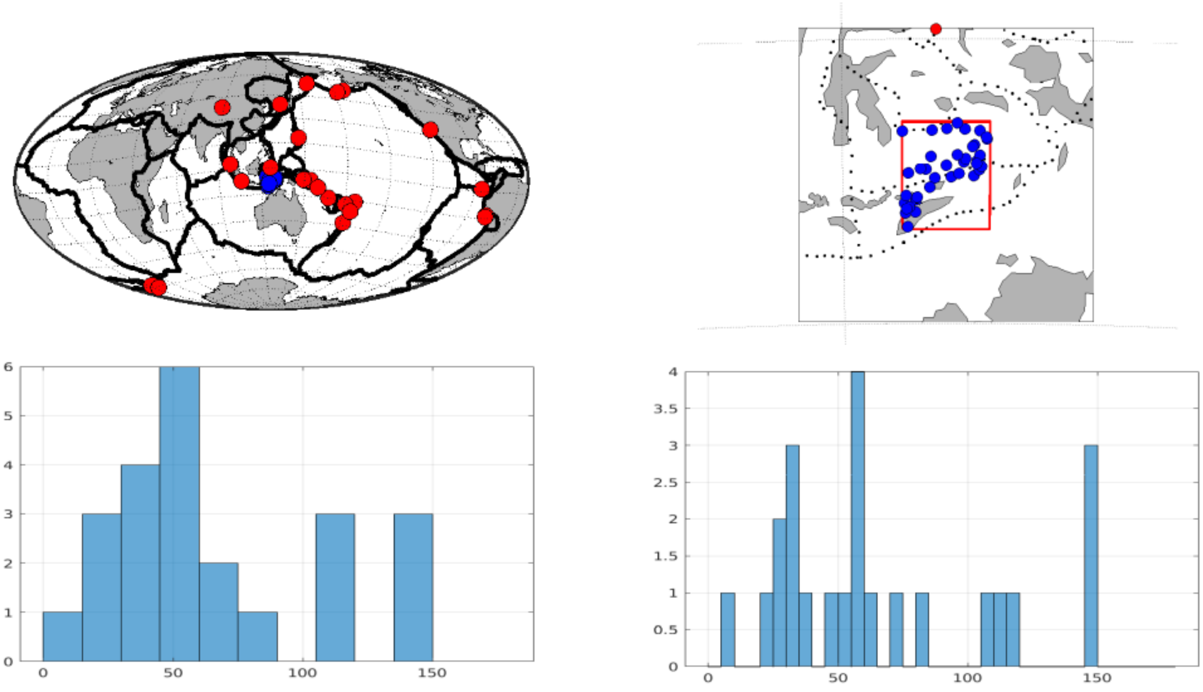

Figure S9: Source event distribution for Figure 2c (text)

Figure 2c indicates 14 potentially triggered events. Those 14 events were all preceded by one or more source events (source events are in red and triggered events are in blue). Locations shown in the top panel, with a global map on the left and the study area on the right. Spatial distributions are shown in the bottom panels (15-degree binning on the left, and 5-degree binning on the right).

Supplemental References

1. Shearer, Peter M., and Philip B. Stark. "Global risk of big earthquakes has not recently increased." *Proceedings of the National Academy of Sciences* 109.3 (2012): 717-721.
2. Hough, Susan E. "Do large (magnitude≥ 8) global earthquakes occur on preferred days of the calendar year or lunar cycle?." *Seismological Research Letters* 89.2A (2018): 577-581.
3. Wyss, Max, and David C. Booth. "The IASPEI procedure for the evaluation of earthquake precursors." *Geophysical Journal International* 131.3 (1997): 423-424.
4. O'Malley, Robert T., et al. "Evidence of systematic triggering at teleseismic distances following large earthquakes." *Scientific reports* 8.1 (2018): 1-12.
5. MATLAB Release 2020b, The MathWorks, Inc, Natick, Massachusetts, United States. https://www.mathworks.com.
